# LK-DFBA: a linear programming-based modeling strategy for capturing dynamics and metabolite-dependent regulation in metabolism

**DOI:** 10.1186/s12859-020-3422-0

**Published:** 2020-03-02

**Authors:** Robert A. Dromms, Justin Y. Lee, Mark P. Styczynski

**Affiliations:** School of Chemical & Biomolecular Engineering, Georgia Institute of Technology, Atlanta, GA USA

**Keywords:** Flux balance analysis, Metabolomics, Metabolic modeling, Metabolite dynamics

## Abstract

**Background:**

The systems-scale analysis of cellular metabolites, “metabolomics,” provides data ideal for applications in metabolic engineering. However, many of the computational tools for strain design are built around Flux Balance Analysis (FBA), which makes assumptions that preclude direct integration of metabolomics data into the underlying models. Finding a way to retain the advantages of FBA’s linear structure while relaxing some of its assumptions could allow us to account for metabolite levels and metabolite-dependent regulation in strain design tools built from FBA, improving the accuracy of predictions made by these tools. We designed, implemented, and characterized a modeling strategy based on Dynamic FBA (DFBA), called Linear Kinetics-Dynamic Flux Balance Analysis (LK-DFBA), to satisfy these specifications. Our strategy adds constraints describing the dynamics and regulation of metabolism that are strictly linear. We evaluated LK-DFBA against alternative modeling frameworks using simulated noisy data from a small in silico model and a larger model of central carbon metabolism in *E. coli*, and compared each framework’s ability to recapitulate the original system.

**Results:**

In the smaller model, we found that we could use regression from a dynamic flux estimation (DFE) with an optional non-linear parameter optimization to reproduce metabolite concentration dynamic trends more effectively than an ordinary differential equation model with generalized mass action rate laws when tested under realistic data sampling frequency and noise levels. We observed detrimental effects across all tested modeling approaches when metabolite time course data were missing, but found these effects to be smaller for LK-DFBA in most cases. With the *E. coli* model, we produced qualitatively reasonable results with similar properties to the smaller model and explored two different parameterization structures that yield trade-offs in computation time and accuracy.

**Conclusions:**

LK-DFBA allows for calculation of metabolite concentrations and considers metabolite-dependent regulation while still retaining many computational advantages of FBA. This provides the proof-of-principle for a new metabolic modeling framework with the potential to create genome-scale dynamic models and the potential to be applied in strain engineering tools that currently use FBA.

## Background

Metabolism is the biochemical supply chain for all other cellular processes, such as DNA replication, transcription of RNA, and protein synthesis. It is perhaps the most immediate readout available of cellular state. An increasing focus on systems-level behavior in cellular biology coupled with the development of appropriate chemical analyses to enable studies of metabolism has led to the field of metabolomics, which measures metabolic intermediates (metabolites) at the systems scale [1].

As a direct readout of the state of cellular metabolism, metabolomics is a natural complement to efforts in metabolic engineering, in which an organism is genetically engineered to facilitate the overproduction of a target small molecule [2]. Some metabolic engineering targets are known products or byproducts of primary metabolism in commonly used model organisms; others derive from secondary metabolism in organisms that may be difficult to culture and thus can be produced in a more cost-efficient manner by exporting the corresponding metabolic pathway into a more amenable host, such as *Bacillus subtilis*, *Escherichia coli* or *Saccharomyces cerevisiae* [3, 4].

Given how tightly tied metabolism is to so many other cellular processes and the fact that some metabolites that are necessary intermediates in metabolism can actually have inherent toxicity to the cell, the metabolic reaction network is highly connected and tightly regulated [5, 6]. Metabolic modeling and computational strain design tools are valuable methods for metabolic engineers to deal with these interactions, allowing them to more strategically allocate the significant time and resources required to produce an engineered strain in the lab.

The primary methods for metabolic engineering strain modeling are constraint-based models (CBMs), of which Flux Balance Analysis (FBA) is the prototypical example [7, 8]. In FBA, the stoichiometry of the metabolic reaction network is combined with an assumption of metabolic steady-state, meaning that for all metabolites their rate of depletion equals their rate of consumption and thus differential equations are not necessary to model the system. This simplified model, combined with restrictions on rates of enzyme reversibility and saturation as well as an objective function describing the cell’s preferred behavior, specify a linear program (LP) [7], which is a tractable optimization problem with extensive prior literature for theoretical and algorithmic treatments. From this, an optimal metabolic flux distribution can be calculated with relatively few data requirements. Due to its simplicity over models based on ordinary differential equations (ODEs) (which involve complex reaction equations and many parameters) and the range of potential modifications, FBA has been the basis for a host of tools for strain design, including OptKnock [9] and its derivatives [10–14]. Complementing these efforts, a great amount of work has gone into genome-scale model reconstructions of many organisms critical to metabolic engineering [15].

However, FBA was developed well before the advent of metabolomics, and some of its core assumptions preclude directly and fully integrating metabolomics data into the model. Other types of data, such as proteomics, transcriptomics, and gene expression data represented as Boolean networks, have been successfully integrated into FBA using various approaches [16–19]. However, the steady-state assumption of FBA, which asserts that metabolite levels do not change over time, removes metabolite concentrations from the model in favor of a computationally convenient linear system of fluxes and network stoichiometry, leaving few clear routes for exploitation of metabolomics data in model development or parameterization. While metabolomics data have been used to estimate reaction feasibility via thermodynamic constraints, direct integration of metabolite concentrations into model development remains largely in the realm of kinetic models, which have the disadvantage of long computation times and difficulties in defining appropriate functional forms for reaction terms, making kinetic models difficult to build at the genome scale [20, 21].

Moreover, the steady-state assumption also complicates the tracking of metabolic dynamics (the changes of metabolite concentrations and reaction fluxes as a function of time due to biochemical and regulatory changes in the cell) because one expects extracellular concentrations and the state of the organism to vary temporally for many industrially and ecologically relevant growth conditions [22, 23]. Information about metabolite concentrations can lead to improvements in metabolic engineering [24] and inform researchers which strain designs are more likely to be feasible (e.g. based on metabolite availability or the level of toxic metabolites). Additionally, the lack of metabolite concentration representation in FBA models also precludes the incorporation of metabolite-level regulation in the model, which is known to have a major impact on metabolic dynamics [25]. Some previously published FBA methods have used metabolite concentrations to better constrain flux values [26, 27] or compartmental modeling to approximate temporal variations [28], but these approaches are still limited to steady-state flux distributions and cannot model full dynamics or regulation. Recently, Moxley et al. combined CBM and kinetic modeling together, which allowed for integration of concentration data and tracking of metabolite dynamics, but their approach is ultimately still an ODE-based framework [29]. Alternatively, the Dynamic Optimization Approach (DOA) of Dynamic Flux Balance Analysis (DFBA) is an extension of FBA that discards the steady-state assumption and adds non-linear constraints, such as those describing batch growth kinetics or kinetic rate laws [30]. However, because of the many non-linear constraints involved, FBA’s most attractive mathematical properties are lost, as linear programs like FBA are a well-understood convex optimization problem that can be solved quite efficiently. The Static Optimization Approach (SOA) of DFBA avoids this issue by removing any non-linear constraints and only driving metabolic dynamics through rate of change of flux constraints. Values for the maximum rate of change of flux are difficult to find in literature, and the parameters were estimated using transcription and translation rates (and thus inherently ignore multiple types of post-transcriptional and post-translational regulation known to be important in metabolism). While the SOA approach retains an LP structure, it cannot incorporate available kinetic or regulatory information as in the DOA approach; overcoming this limitation could improve model accuracy and provide more insight on the dynamic behavior of the system. Table 1 summarizes the advantages and disadvantages of each aforementioned modeling approach.

**Table 1:** Advantages and disadvantages of several constraint-based and ODE-based models

Method	Advantages	Disadvantages
FBA	Efficient to solve for steady-state fluxes; many metabolic engineering tools built around FBA	Cannot track metabolite dynamics
ODE-based models	Able to track metabolite dynamics; accurately model reaction kinetics	Abundant kinetic parameters; difficult to scale up to large systems
DFBA (DOA)	Able to track metabolite dynamics; does not require complex kinetic equations for each flux	Contains non-linear constraints that can make the optimization problem difficult to solve
DFBA (SOA)	Able to track metabolite dynamics; does not require non-linear constraints	Cannot incorporate kinetic or regulatory information

In this work, we modified the DFBA formulation with the goals of allowing the integration of metabolomics data and capturing metabolite-level regulation and dynamics while still maintaining an LP structure. Kinetics and regulation are approximated from metabolomics data as a set of linear equations specifying upper bounds on flux values, which in turn drive metabolite dynamics. These equations are applied over the discretized simulation interval and represent kinetic expressions similar to the constraints found in the DOA approach of DFBA, but are completely linear and are combined with the other elements of FBA to form an LP problem that can be solved as efficiently as the SOA approach [30]. The result, which we call Linear Kinetics-Dynamic Flux Balance Analysis or LK-DFBA, is a system that combines the main advantages of the DOA and SOA approaches of DFBA, and can be directly combined with any of the strain design tools that work with FBA models as their input. The linear structure of LK-DFBA provides the potential for our framework to be eventually applied at the genome scale (a task that is especially problematic for ODE-based models) while continuing to track metabolite dynamics.

As a first demonstration of the proof-of-principle for such an approach, we explored this framework in two model systems of varying scale, generating in silico reference time course data and noisy synthetic time course data by varying the data sampling frequency and the coefficient of variance (CoV) of added noise. We compared our approach to ODE-based frameworks that use Biochemical Systems Theory (BST) power-law kinetics and Michaelis-Menten (MM) rate laws, finding that LK-DFBA is able to capture the behavior of the original model systems and can outperform the BST-based comparator under the conditions most relevant to metabolomics data (low sampling frequency and high noise). We also explored some challenges associated with model scale-up and structural features unique to the different model systems. Finally, we briefly discuss some of the next steps that could lead to LK-DFBA becoming a widely-adopted approach for modeling metabolic systems.

## Methods

### Simulating regulated metabolite dynamics with a linearly-constrained program

LK-DFBA takes as input two sets of information. The first set comprises the constraints and objective from FBA: a stoichiometric matrix describing the relationship between metabolites and fluxes in the model, a set of upper and lower bounds on metabolic fluxes, and an objective function specifying the flux(es) the network tries to locally maximize or minimize. To these, we add metabolite concentration initial conditions, a simulation time interval, a parameter describing the number of segments into which the interval is to be evenly divided, and a list of regulatory interactions (and the corresponding parameters to describe them). These elements are described in more detail in Fig. S1 and the Methods S1 in the Supplementary Information (Additional file 1).

#### The solution vector

In LK-DFBA, we relax the steady-state assumption, working from

1 $$ \frac{\mathrm{d}\overset{\rightharpoonup }{\mathrm{x}}}{\mathrm{d}\mathrm{t}}=\mathrm{S}\overset{\rightharpoonup }{\mathrm{v}}={\overset{\rightharpoonup }{\mathrm{v}}}_{\mathrm{p}} $$

where S is the n_m_ × n_v_ stoichiometric matrix from FBA, $\overset{\rightharpoonup }{\mathrm{v}}$ is the flux distribution from FBA, and $\frac{\mathrm{d}\overset{\rightharpoonup }{\mathrm{x}}}{\mathrm{d}\mathrm{t}}$ is equivalent to the vector of “pooling fluxes” (using the nomenclature of Covert et al. in iFBA [31]) for metabolites, such that v_p,i_ corresponds to changes in x_i_. Rearranging the ${\overset{\rightharpoonup }{\mathrm{v}}}_{\mathrm{p}}$ term and combining it with the original solution vector term $\overset{\rightharpoonup }{\mathrm{v}}$ gives us

2 $$ 0=\mathrm{A}\overset{\rightharpoonup }{\mathrm{w}}=\left[\mathrm{S}\kern0.5em -\mathrm{I}\right]\left[\begin{array}{c}\overset{\rightharpoonup }{\mathrm{v}}\\ {}{\overset{\rightharpoonup }{\mathrm{v}}}_{\mathrm{p}}\end{array}\right] $$

where A is the (n_m_ × (n_m_ + n_v_)) augmented stoichiometric matrix and $\overset{\rightharpoonup }{\mathrm{w}}$ is the ((n_m_ + n_v_) × 1) augmented flux vector. Combining the augmented flux vector over each time segment and the concentrations at each time point, the final solution vector for the LP is constructed as

3 $$ \upomega ={\left[{\overset{\rightharpoonup }{\mathrm{w}}}^{\mathrm{T}}\left({\mathrm{t}}_1\right),\dots, {\overset{\rightharpoonup }{\mathrm{w}}}^{\mathrm{T}}\left({\mathrm{t}}_{\mathrm{nT}}\right),{\overset{\rightharpoonup }{\mathrm{x}}}^{\mathrm{T}}\left({\mathrm{t}}_0\right),{\overset{\rightharpoonup }{\mathrm{x}}}^{\mathrm{T}}\left({\mathrm{t}}_1\right),\dots, {\overset{\rightharpoonup }{\mathrm{x}}}^{\mathrm{T}}\left({\mathrm{t}}_{\mathrm{nT}}\right)\right]}^{\mathrm{T}} $$

and is of dimension ((n_v_ + n_m_) ∙ nT + n_m_ ∙ (nT + 1) × 1), where n_v_ is the number of system fluxes, n_m_ is the number of metabolites, and nT is the number of time intervals into which the simulation period has been discretized.

#### Linearized kinetics constraints

The key feature of LK-DFBA is the addition of linear equations to describe constraints in which fluxes are controlled by metabolites, as is the case in circumstances ranging from mass action kinetics to allosteric regulation (on short time scales) or transcriptional regulation (on longer time scales). Any dependence of flux on metabolite concentrations is implemented in this manner, a critical element for driving biologically relevant dynamics in the model.

These constraints are specified by a list of n_r_ mappings. Corresponding to each mapping is a pair of parameters (*a*, *b*) such that for mapping *n* between “controller” metabolites {*x*}_*n*_ and “target” fluxes {*v*}_*n*_,

4 $$ \sum \limits_i{v}_{i,n}\left({t}_{k+1}\right)\le {a}_n\left(\sum \limits_j{x}_{j,n}\left({t}_k\right)\right)+{b}_n, $$

where *v*_*i*,*n*_ is a target flux in {*v*}_*n*_, *x*_*j*,*n*_ is a controller metabolite in {*x*}_*n*_, and (*a*_*n*_, *b*_*n*_) are the parameters describing the linear kinetics constraint. When *a*_*n*_ > 0, this interaction produces a promotional effect, and when *a*_*n*_ < 0, this interaction has an inhibitory effect.

To perform a given simulation, the set of (*a*, *b*) parameter values is provided along with the list of controller and target mappings. In practice, this will need to be determined via parameter fitting, as the linear equations in general are simplified approximations that do not directly correspond to intrinsic physical quantities. We discuss these constraints and their parameterization further in the Methods S1, Additional file 1. In addition to linear kinetics constraints, fluxes are also constrained by lower and upper bounds, as in FBA. While arbitrary, large nominal values can be used as upper bounds, known values can also be used to represent saturation.

#### The LK-DFBA optimization problem

Assembling constraints produces the following linearly-constrained quadratic program (QP) (penalizing the solution vector norm) for simulating metabolic time courses which we refer to as LK-DFBA. For

$$ \overset{\rightharpoonup }{\omega }={\left[{\overset{\rightharpoonup }{w}}^T\left({t}_1\right),\dots, {\overset{\rightharpoonup }{w}}^T\left({t}_{nT}\right),{\overset{\rightharpoonup }{x}}^T\left({t}_0\right),{\overset{\rightharpoonup }{x}}^T\left({t}_1\right),\dots, {\overset{\rightharpoonup }{x}}^T\left({t}_{nT}\right)\right]}^T, $$

5 $$ {\mathit{\max}}_{\overset{\rightharpoonup }{\omega }}\ z={\overset{\rightharpoonup }{c}}^T\overset{\rightharpoonup }{\omega }-\lambda {\overset{\rightharpoonup }{\omega}}^T\overset{\rightharpoonup }{\omega } $$

$$ \left(\mathrm{s}.\mathrm{t}\right)\kern0.5em 0=A\overset{\rightharpoonup }{w}\left({t}_k\right)\kern1em \forall k\in \left[1, nT\right] $$

$$ {\overset{\rightharpoonup }{w}}_{LB}\le \overset{\rightharpoonup }{w}\left({t}_k\right)\le {\overset{\rightharpoonup }{w}}_{UB}\kern1em \forall k\in \left[1, nT\right] $$

$$ {\overset{\rightharpoonup }{x}}_{LB}\le \overset{\rightharpoonup }{x}\left({t}_k\right)\le {\overset{\rightharpoonup }{x}}_{UB}\kern1em \forall k\in \left[1, nT\right] $$

$$ \overset{\rightharpoonup }{x}\left({\mathrm{t}}_0\right)={\overset{\rightharpoonup }{x}}_0 $$

$$ {x}_i\ \left({t}_k\right)={x}_i\ \left({t}_{k-1}\right)+\varDelta t\bullet {v}_{p,i}\ \left({t}_k\right)\kern1em \forall k\in \left[1, nT\right] $$

$$ {\sum}_i{v}_{i,n}\left({t}_{k+1}\right)\le {b}_n+{a}_n{\sum}_j{x}_{j,n}\left({t}_k\right) $$

$$ \forall k\in \left(1, nT\right),\forall i\in {\left\{v\right\}}_n,\forall j\in {\left\{x\right\}}_n,\forall n\in \left(1,{n}_r\right) $$

### Model generation code

We developed MATLAB code to automatically translate a standard FBA model into an LK-DFBA model and solve the resulting optimization problem using the Gurobi Optimizer [32]. This code has been made publicly available on GitHub at https://github.com/gtStyLab/lk-dfba.

### Test models

#### The branched pathway model

**Fig. 1:**
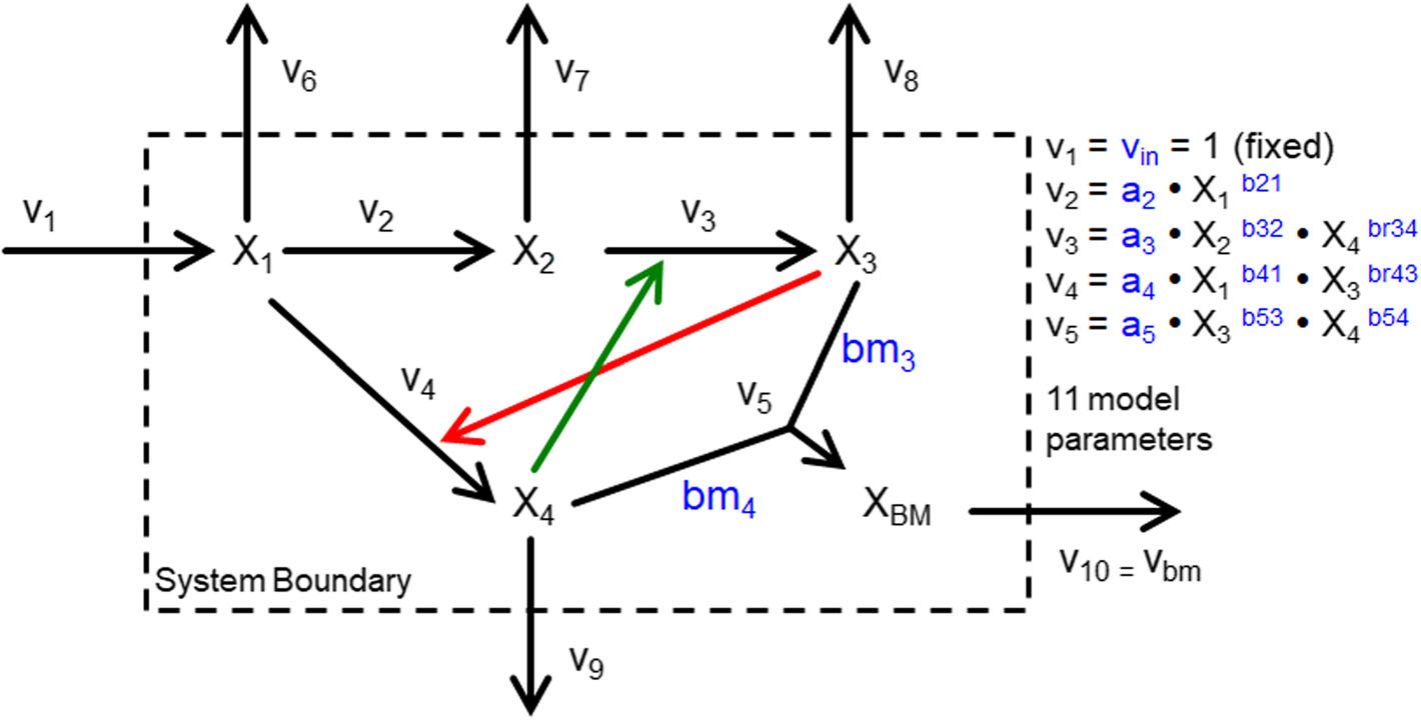
The modified branched pathway model used in this work, adapted from Voit and Almeida [33]*.* Black arrows indicate fluxes. The green and red arrows denote positive and negative regulatory interactions, respectively. The dashed line denotes the system boundary. Metabolites are X_1_, X_2_, X_3_, X_4_, and X_BM_. System fluxes are v_1_, v_2_, v_3_, v_4_, and v_5_. Pooling fluxes for X_1_, X_2_, X_3_, X_4_, and X_BM_ are v_6_, v_7_, v_8_, v_9_, and v_BM_, respectively. The parameters in blue specify reaction rates and stoichiometry, such that *bm*_3_*X*_3_ + *bm*_4_*X*_4_ → *X*_*BM*_. Not shown are initial conditions. Kinetic rate laws are implemented as generalized mass action (GMA) rate laws from BST

Our first test model is a modified version of a popular, well-established in silico model from BST [33] describing a simple branched pathway with both positive and negative regulatory interactions; it is shown in Fig. 1. As in the original BST model, we use power-law kinetics. Parameterizations for this model are shown in Table S1, Additional file 1.

#### Glycolysis and pentose phosphate pathway in *E. coli*

To explore initial scale-up and to better gauge the challenges of implementing LK-DFBA, we tested a model of central carbon metabolism in *E. coli* comprising glycolysis and the pentose phosphate pathway (PPP) with empirically derived rate laws [34, 35]. The network structure is shown in Fig. S2, with a list of model abbreviations in Table S2 and S3 in the Supplementary Information (Additional file 1). Because LK-DFBA retains a linear structure, the framework could potentially be applied to much larger systems if the stoichiometry and regulation of the system are known, and if metabolomics data from the system are available. Noiseless data at high resolution were generated [36] from the default model initial conditions and parameters in COPASI 4.14 (Build 89), with the exception that moieties such as ATP, ADP, and NADH, etc. were held at constant concentrations during simulation [34, 35, 37, 38].

### Generating noise-added datasets

We generated datasets with different sampling frequencies and noise characteristics using a previously described procedure, allowing us to produce multiple replicates of noisy data with a specified sampling frequency and measurement noise [36].

Briefly, the noiseless data at high sampling frequency were down-sampled such that the initial conditions and nT additional time points are sampled evenly over the time interval of interest. Then, the metabolite or flux values are replaced with a random value drawn from *N*_*i*,*k*_~(*y*_*i*_(*t*_*k*_), *CoV* ∙ *y*_*i*_(*t*_*k*_)), where *y*_*i*_(*t*_*k*_) is the value of species (metabolite or flux) *i* at time point *k*, and CoV is the coefficient of variance. We leave the initial conditions at the original values from the source model, and use it as unfitted input for the LK-DFBA simulation.

### Parameter fitting

We pose the parameter-fitting problem as follows: given data describing a set of metabolite (and flux) time courses, determine the set of model parameters that minimize the weighted sum of squares error between the data and the time courses predicted by the model. We explored several strategies for addressing this problem. For all methods, we assumed that the structure of the network and the regulatory interactions were known, including the signs of the interactions. In all cases, the true initial conditions (i.e. with no noise added) were provided for all metabolites.

#### Dynamic flux estimation and parameter regression

**Fig. 2:**
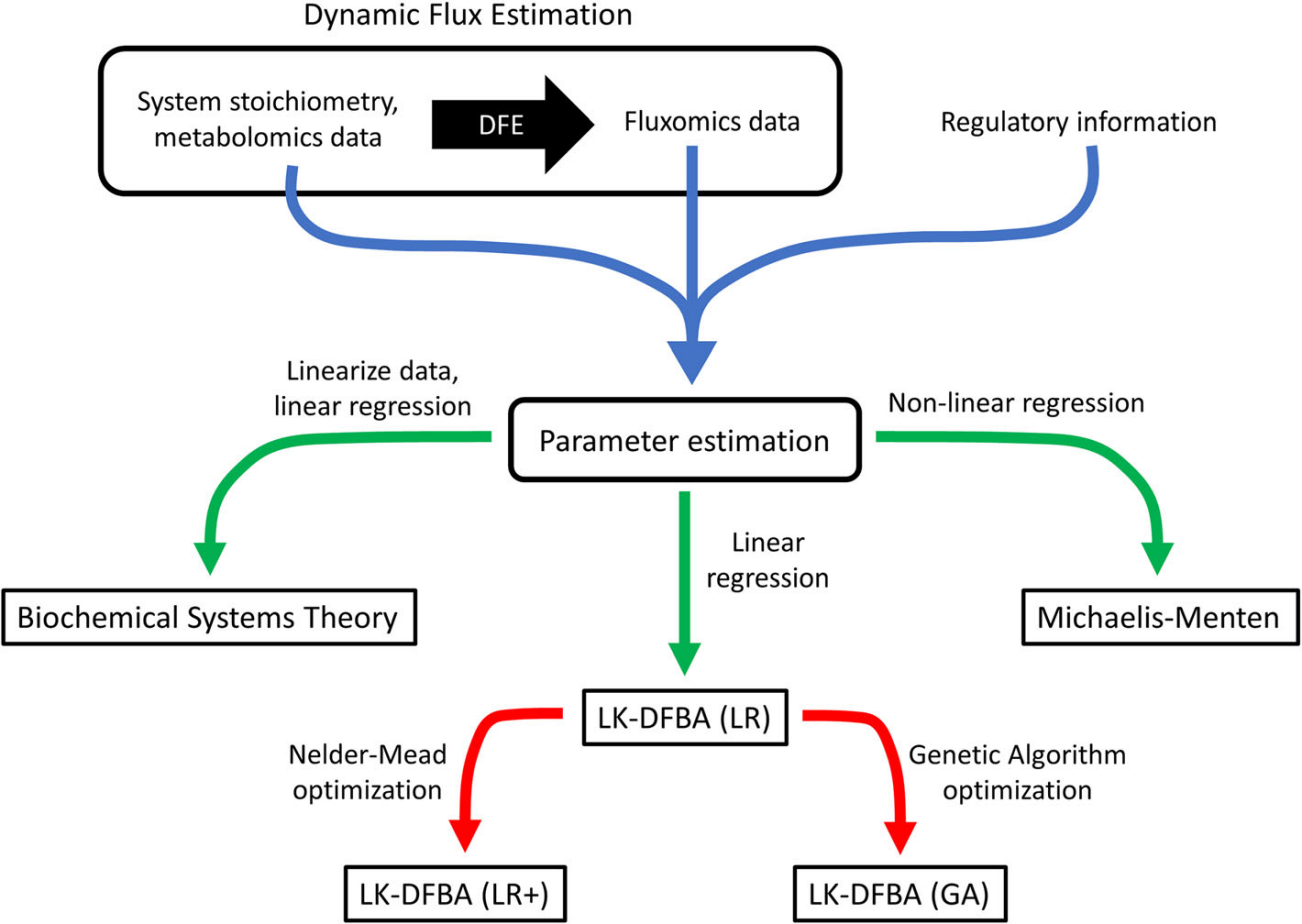
Workflow for building metabolic models. Dynamic Flux Estimation is applied to the system stoichiometry and available metabolomics data to infer flux data. The system stoichiometry, metabolomics data, inferred flux data, and system regulatory information are then used to estimate parameters in each modeling framework (blue arrow), using linear or non-linear regression (green arrows). A secondary optimization step can be applied after LK-DFBA (LR) to further improve modeling accuracy (red arrows)

Figure 2 presents a workflow for building each model. We started with a Dynamic Flux Estimation (DFE) scheme to fit noisy data and infer flux data [39]. We smoothed concentration time course profiles using an impulse function as previously described [36], and determined the slope (metabolite accumulation or pooling flux) from the derivative of the smoothed function. From these slope values the dynamic flux distribution was calculated according to a procedure based on the method of Ishii et al. [38]. Fluxes were divided into “static” and “dynamic” sets, and the stoichiometric mass balance equations were re-organized to solve for the “dynamic” fluxes using MATLAB’s backslash pseudo-inverse. From this, we paired the resulting calculated dynamic flux distribution data with the original noisy concentration data, system stoichiometry, and regulatory information for subsequent regression analysis (blue arrow in Fig. 2).

To estimate model parameters in the two ODE-based approaches (‘BST’,‘MM’) and our new approach (‘LK-DFBA (LR)’), we used linear or non-linear regression on the inferred flux data and the concentration data to fit the parameters of the individual rate law equations to the corresponding flux and metabolite data (green arrows in Fig. 2). For the BST-based generalized mass action kinetic rate law model (‘BST’), we log-transformed the data to linearize the system and solved for the power-law parameters using linear regression. For the Michaelis-Menten kinetic rate law model (‘MM’), we performed a non-linear regression by seeding the solver with 100 random initial parameters and selecting the fit with the lowest residuals. For the Linear Regression LK-DFBA model (LK-DFBA (LR)), we performed linear regression on the combined flux and concentration data for each target-controller mapping as appropriate (for example, regression on the sum (v_2_ + v_4_) against X_1_ when controller metabolite X_1_ is mapped to target fluxes v_2_ and v_4_).

#### Parameter optimization

To fit parameters using a non-linear optimization approach (red arrows in Fig. 2), we constructed a fitness function from the weighted sum-of-squares error (SSE) between the provided data and model predictions, subject to an L_2_ regularization penalty on the fitted parameters. The SSE weights are specified by the user, and we used them to reflect features such as differences in scale between metabolites. For the “Linear Regression-Plus” method (‘LKDFBA-(LR+)’), we used the results of the Linear Regression (‘LK-DFBA (LR)’) method (described above) as an initial starting point for the Nelder-Mead simplex solver using the MATLAB function fminsearch() to fit an LK-DFBA model. We also tested a global optimization approach using a genetic algorithm (Methods S1, Additional file 1) but found the LK-DFBA (LR+) method to be much less computationally expensive with similar accuracy (Fig. S8, Additional file 1).

#### Missing metabolite time courses

During our analysis, we explore the impact of incomplete data in the form of missing time course data. To model this, we select a metabolite, designate it as “missing,” and withhold the time course data for that metabolite from the analysis (with the exception that we provide the initial concentration of the metabolite as a means of starting the process). For the DFE procedure, we designate the pooling flux as a static flux and set its value to 0 on the basis that we have no information to justify assigning it a non-zero value. Similarly, the weight of this metabolite is set to 0 in the fitness function to preclude it from influencing parameter optimization.

#### Assessing fitted model performance: metrics and equations

For each fit, we calculated the penalized relative SSE (prSSE) to allow us to compare each modeling method based on the conditions used to generate the noisy synthetic data (CoV, nT, missing X_i_).

First, for model *m* and noisy data replicate *n*, we calculate the resulting simulated time course data as

6 $$ \tilde{y}_{j,k,m,n}={f}_m\left({\overset{\rightharpoonup}{\mathrm{x}}}_n^0;{\theta}_{m,n}\right) $$

where $\tilde{y}_{j,k,m,n}$ is the simulated value of concentration or flux *j* at time *k* for model *m* fitted to noisy data replicate *n*, and *f*_*m*_ is the function integrating model *m* over the time course with initial conditions ${\overset{\rightharpoonup }{x}}_n^0$ and fitted parameters *θ*_*m*,*n*_. From this, we calculated prSSE as

7 $$ {prSSE}_{m,n}={w}_{m,n}{\sum}_j{w}_j\frac{\sum_{k=1}^{n_k}{\left(\tilde{y}_{j,k,m,n}-{y}_{j,k}\right)}^2}{n_k} $$

where *y*_*j*,*k*_ is the value of species *j* at time *k* in the original noiseless time course data, *n*_*k*_ is the number of time points in the simulation interval,

8 $$ {w}_j={w}_{\ast }(j)\ {\left(\mathit{\max}\left({\overset{\rightharpoonup }{y}}_j\right)-\mathit{\min}\left({\overset{\rightharpoonup }{y}}_j\right)\right)}^{-1} $$

is the species scaling factor, ${\overset{\rightharpoonup }{y}}_j$ is the noiseless data time course for species *j*, *w*_∗_(*j*) is a binary variable denoting participation in the prSSE calculation (e.g. for *j ϵ* pooling fluxes, we set *w*_∗_(*j*) to 0 to exclude them from the prSSE),

9 $$ {w}_{m,n}={\left(\frac{n_f(m)\bullet nT(n)-{n}_p(m)}{n_f(m)\bullet nT\left(\mathrm{n}\right)}\right)}^{-1} $$

is the penalty on parameterization, *n*_*f*_(*m*) is the number of species used to fit *θ*_*m*,*n*_, *nT*(*n*) is the number of time points used to fit *θ*_*m*,*n*_, and *n*_*p*_(*m*) is the number of parameters in *θ*_*m*,*n*_.

## Results

### Comparing the performance of methods using noisy data in the branched pathway model

We generated high-resolution noisy time course data for the modified branched pathway model using the k = 1 parameter set by downsampling the high resolution data to nT = 15, 20, 30, 40, and 50, and adding multiplicative Gaussian noise to data points after the initial time point with CoV = 0.05, 0.15, and 0.25. For each combination of nT and CoV, 50 replicate datasets were produced, producing a total of 750 noisy datasets.

**Fig. 3:**
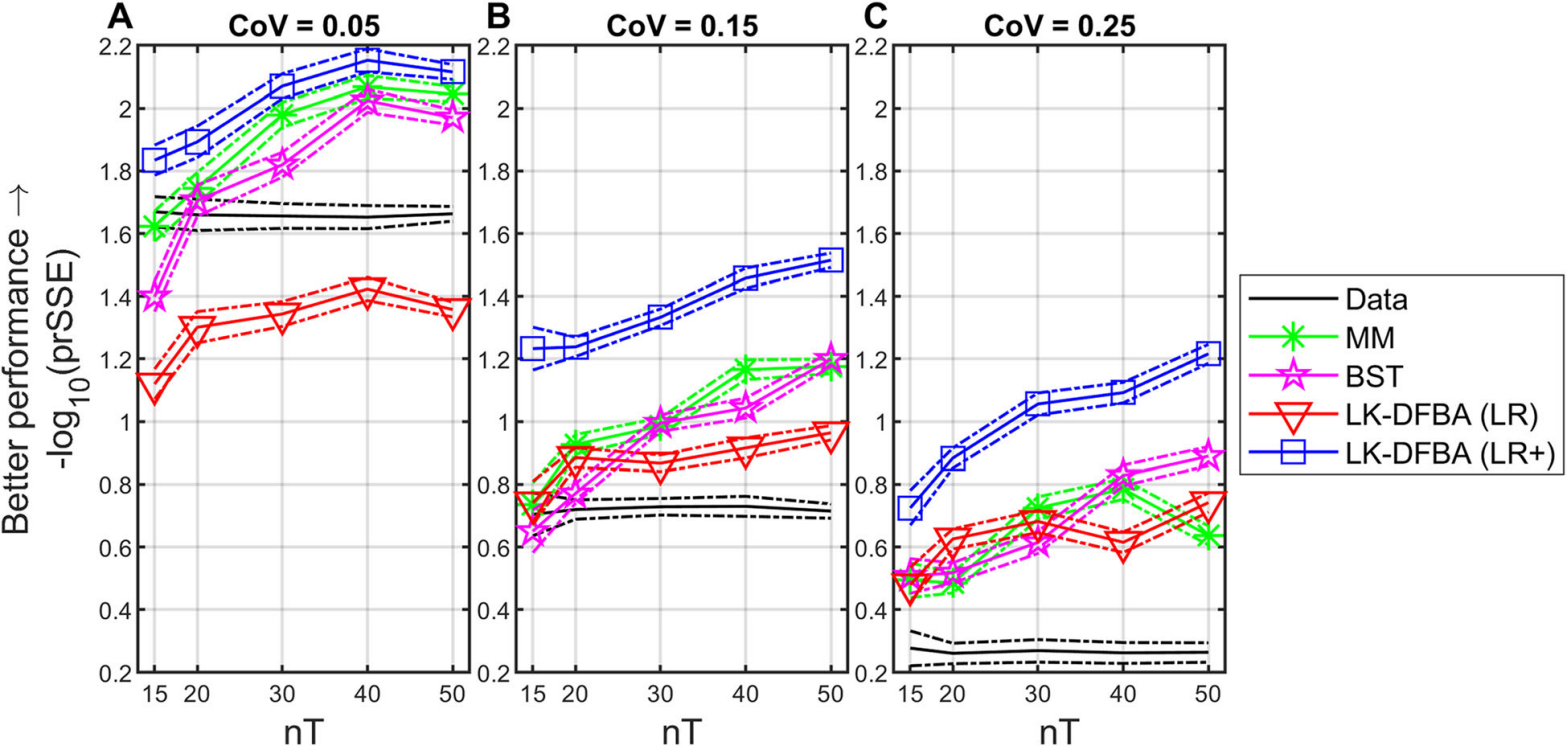
Comparison of fitting performance for MM, BST, LK-DFBA (LR), and LK-DFBA (LR+) methods. The black line for data is a benchmark comparison reflecting the noise added to the input data: each of the 750 noisy datasets was compared against the noise-free data to establish a baseline level of inaccuracy dependent on CoV. The penalized relative sum-of-square error (prSSE) calculations terms are all normalized to allow a consistent comparison against this reference. Solid lines represent the median error for each modeling approach and dotted lines represent the median absolute deviation. **a** CoV = 0.05. **b** CoV = 0.15. **c** CoV = 0.25. nT is the number of time points used to fit each model. The LK-DFBA framework with LK-DFBA (LR+) performs particularly well compared to other approaches when input data have significant noise, which is the type of input to be expected from experimental metabolomics analyses

For each noisy dataset replicate and each modeling method (MM, BST, LK-DFBA (LR), LK-DFBA (LR+)) we employed the corresponding fitting procedures described in the Methods. Sensitivity analysis of the LK-DFBA (LR+) parameters indicates that there are several key constraints that have stable parameters across the different combinations of nT and CoV, while other parameters are more flexible (Fig. S11, Additional file 1). The fitted parameters were used to simulate the system time course for each case at high resolution (nT = 200), and the fitted models were compared against the noiseless version of the data to calculate model prSSE as described in the Methods section. The results of this analysis are shown in Fig. 3.

In this analysis, we observe a few basic trends. First, as expected, as the quantity of data in the time courses increase, the methods all consistently achieve lower error (higher -log10(prSSE)), with some evidence of diminishing returns in a few cases at high nT. In addition, the quality of fits decreases as the added noise increases. Across conditions, the LK-DFBA (LR+) method outperforms all other methods. We also note that the BST and MM methods generally perform well in cases when data quality is very good, such as at low noise (CoV = 0.05), or when there is a high sampling rate (nT = 40, nT = 50). When data are more sparse or noisy, the LK-DFBA (LR) method performs comparably or slightly better than the BST and MM methods. Surprisingly, the MM method performs better than the BST method at low noise (CoV = 0.05), but the two methods are comparable at higher noise levels. We note that like the improvement from LK-DFBA (LR) to LK-DFBA (LR+), an additional parameter optimization step for the BST model can produce better results for this model as well; however, this improved performance (in which it outperforms LK-DFBA (LR+), except in metabolomics data with low sampling frequency and high noise) is to be expected given that the BST model has the advantage of containing the true underlying system structure and kinetic rate laws, even though that would not be true for a real biological system. This is further illustrated in Fig. S8, Additional file 1 where the BST model outperforms all other frameworks on noiseless data. For these reasons, the BST model was included as a best-case scenario for situations (discussed below) where a metabolite time course is missing, while the MM method was omitted. No additional parameter optimization step was tested with the MM approach because the solver in the original parameter estimation is already seeded with 100 random initial parameters to help avoid getting trapped in local minima.

### The effects of withholding metabolite time courses on model performance in the branched pathway model

**Fig. 4:**
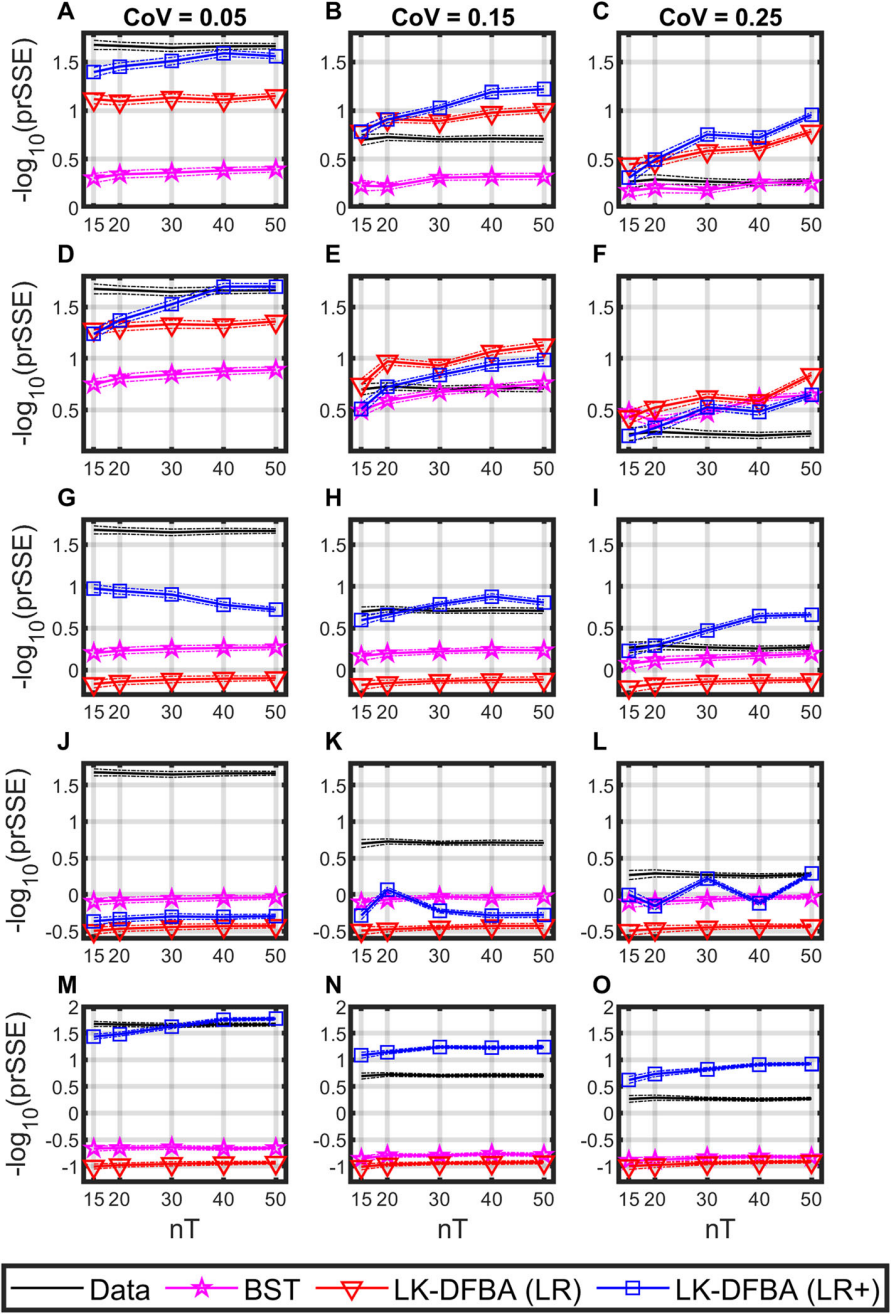
Comparison of fitting performance when one metabolite time course is withheld from the fitting procedure. Solid lines represent the median penalized relative sum-of-square error (prSSE) for each modeling approach and dotted lines represent the median absolute deviation. **a, b, c** Performance when X_1_ is missing (X_1_-Missing). **d, e, f** X_2_-Missing. **g, h, i** X_3_-Missing. **j, k, l** X_4_-Missing. **m, n, o** X_BM_-Missing. nT is the number of time points used to fit each model. Generally, LK-DFBA (LR+) performs better than the compared methods, with the exception of decreased performance of LK-DFBA when the metabolite regulator X_4_ is missing, reflecting the importance of being able to measure regulatory molecules

To test the impact of missing metabolite data (which are to be expected in metabolomics experiments) on fitting performance, we repeated the analysis from the previous section, but modified the procedure by withholding information about one metabolite from the fitting pipeline to model it as “missing” from the data (the value of the metabolite’s initial condition was retained). This was accomplished by setting the pooling flux of the missing metabolite as “static” for the flux estimation step, and the corresponding regressions were performed with only the initial value as a placeholder. Each of the five metabolites in the branched pathway were modeled as missing in this way, for each of the 750 noisy datasets from the previous section. For each case, the BST, LK-DFBA (LR), and LK-DFBA (LR+) fitting methods were performed. In the case of the LK-DFBA (LR+) method, the missing metabolite was also removed from the weights of the fitness function. The results of this analysis are shown in Fig. 4.

The position of the missing metabolite in the metabolic network leads to trends that can differ substantially from those in Fig. 3. These trends stem from how well the dynamic flux estimation step can be performed (which is common to all of the modeling approaches being considered); by setting the pooling flux of a missing metabolite as static, the calculated system fluxes adjacent to that metabolite are skewed accordingly. This in turn affects the regression step and the resulting parameters.

While non-linear parameter fitting may be useful for counteracting this source of inaccuracy, it is not guaranteed to do so. An interesting outcome from this analysis is the performance of the LK-DFBA (LR) and LK-DFBA (LR+) methods in Fig. 4e-f, in which the LK-DFBA (LR) method actually outperforms the LK-DFBA (LR+) method. In those X_2_-Missing cases, the lack of data describing X_2_ dynamics led to poor optimization: the parameters that best optimized the remaining data pushed the model to poorly approximating the time course of the unmeasured metabolite (which was still included in the calculation of prSSE) (see Fig. S9, Additional file 1). We also observe that when X_4_ is withheld from flux estimation and parameter optimization, the LK-DFBA (LR+) model usually fails to outperform the BST model (represented in Fig. 4j-k). This suggests that X_4_ has a larger impact on the ability of the LK-DFBA (LR+) model to capture the correct behavior. Given that X_4_ is the controller for one of the two regulatory interactions, this serves to highlight the importance of capturing metabolite dynamics in order to incorporate regulation, and further justifies our interest in modeling these sorts of interactions.

### Recapitulating results with the *E. coli* model

The branched pathway model is useful for exploring some basic characteristics of our new framework, but it lacks biologically relevant features. To introduce some of these complexities and to explore the performance of our approach with a medium-scale model with biological relevance, we generated synthetic data using the kinetic *E. coli* model of Chassagnole et al. [34]. The topology of this network is more complicated, with multiple examples of branch and convergence points that are found throughout genome-scale models. Implementing this model in LK-DFBA resulted in several modifications to our procedure, which are discussed in more detail in the Methods S1, Additional file 1.

We produced synthetic noisy data from the *E. coli* model using the procedure previously described. High-resolution noise-free data for the model’s 18 metabolites and 48 fluxes were generated over the interval of 10s from the ODE model and nominal parameters. From this, we produced 20 noise-added replicates each for nT = 20, 30, and 40 and CoV = 0.10 and 0.20 (for a total of 120 noisy datasets). For these datasets, we encountered significant difficulties in recapitulating a qualitatively correct dynamic flux distribution using impulse smoothing and the procedure of Ishii et al. [38] for dynamic flux estimation (before any LK-DFBA calculations were performed), as shown in Fig. S10, Additional file 1. While DFE works well for determined and overdetermined systems, such as the branched pathway model, a more arduous, systematic approach is necessary for underdetermined systems such as the *E. coli* model [40]. To circumvent this issue and thus focus on assessing the modeling framework itself rather than confounding effects caused by an upstream data analysis procedure, we opted to instead use noise-added flux data directly from the underlying ODE model for regression.

We fit each of these noisy datasets using two different implementations of LK-DFBA to address some complexity present in the *E. coli* model that was not in the branched pathway model. In the *E. coli* model (as in most biological systems), most metabolites can be transformed into multiple potential products, yielding many “branch points” in the model (compared to only one branch point in the previously analyzed model). In the first implementation, a single constraint was used to limit the total efflux from a given metabolite (i.e. all fluxes from a metabolite were listed as targets for that constraint), entailing 18 constraints (36 parameters) of this type. In the second case, we split the targets so that each metabolite-flux mapping had only one target flux, which yielded 49 constraints (98 parameters). We refer to the two model implementations as the “unsplit” and “split” constraint implementations, respectively. For both models, we also included 6 constraints (12 parameters) describing allosteric regulation interactions, resulting in fitting 24 constraints (48 parameters) in the unsplit implementation, and 55 constraints (110 parameters) in the split implementation. The remaining 17 constraints (34 parameters) for the degradation and dilution reactions were modeled as first order kinetic rate laws by setting b = 0 and a = 2.78e-05, corresponding to the ODE model growth rate.

**Fig. 5:**
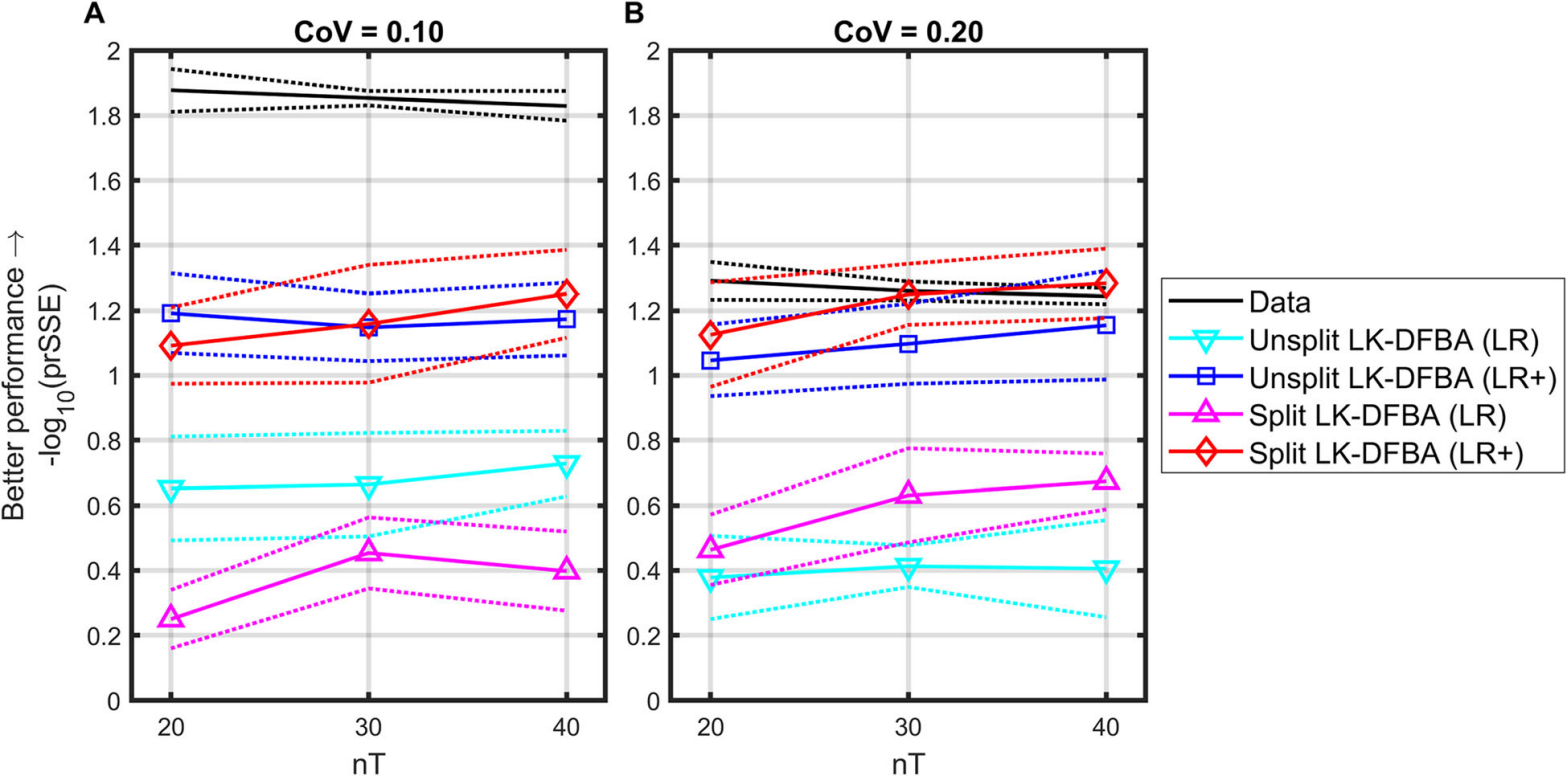
Results of fitting the Unsplit and Split LK-DFBA models to the *E. coli* data. Solid lines represent the median penalized relative sum-of-square error (prSSE) for each modeling approach and dotted lines represent the median absolute deviation. **a** CoV = 0.10. **b** CoV = 0.20. nT is the number of time points used to fit each model. At higher noise and higher data frequency, the split method performs better even when penalized for having additional fitted parameters

We analyzed whether the additional parameters introduced in the split constraints implementation were justified by an improvement in model accuracy, reflected by penalizing the relative SSE value commensurate with the additional parameters when calculating prSSE. For each implementation and noisy dataset, we identified parameters both with the LK-DFBA (LR) and LK-DFBA (LR+) methods, modifying the LK-DFBA (LR+) method to split up and sequentially fit subsets of the parameters (instead of estimating them all simultaneously) as described in the Methods S1, Additional file 1. As with the branched pathway model, we evaluated the quality of the resulting fits by simulating the system at high resolution (nT = 200) and calculating the prSSE. The results of this analysis are shown in Fig. 5.

We note several trends in Fig. 5. First, the unsplit model behaves with the same general trends we observed in the branched pathway model, in which increasing nT and decreasing CoV consistently lead to improved prSSE, and the LK-DFBA (LR+) method outperforms the LK-DFBA (LR) method. Second, the split model generally outperforms the unsplit model (though not always statistically significantly), with the exception of using the LK-DFBA (LR) method on CoV = 0.10 data and LK-DFBA (LR+) on CoV = 0.10 data with nT = 20. Third, the split model performs better on the CoV = 0.20 data than on the CoV = 0.10 data, for both the LK-DFBA (LR) and LK-DFBA (LR+) methods. On average, for the LK-DFBA (LR+) method the unsplit model (48 parameters) took ~ 30 min to fit for each noisy dataset, while the split model (110 parameters) took ~ 50 min (for both split and unsplit models, the LK-DFBA (LR) method took fractions of a second).

## Discussion

In this work, we devised and implemented LK-DFBA, a modification of DFBA that integrates metabolomics data and allows us to capture metabolite dynamics and metabolite-dependent kinetics and regulatory interactions while retaining the linearity of regular FBA. Given the same information necessary for FBA, initial conditions for metabolites, and a suitable description of the connectivity and parameterization of the kinetics interactions, we showed that LK-DFBA successfully reproduces biologically relevant model dynamics in a simplified model system and in a biologically relevant system, competitive with existing approaches that do not have the generalizability to potentially large-scale systems allowed by the linear modeling approach used here. Critically, our approach is more robust than other methods under the most realistic cases (high noise and low sample frequency), which will be crucial for turning metabolomics measurements into biological insight.

The lynchpin of the LK-DFBA framework is the addition of linear kinetics constraints in conjunction with pooling fluxes. On their own, pooling fluxes are not sufficient to induce biologically relevant behavior, and other information (kinetic rate law equality [31] or inequality [30] constraints; connected biological process modules [41]) must be included to drive accumulation and depletion. While the idea of linearized regulation has been implemented before in a CBM of intracellular signal transduction, this approach ignored concentrations and presumed steady-state behavior [42]; the resulting linear constraints simply specified certain flux tradeoffs. In LK-DFBA, we combine both elements to permit and drive metabolite dynamics while preserving the advantages of FBA.

The inclusion of regulatory control driven by metabolic data may enable identification of metabolic regulatory structure in closely related organisms with similar metabolic network topology that otherwise demonstrate highly divergent metabolic dynamics. By retaining the LP structure and the original stoichiometry of the FBA problem, we have created a problem that can integrate metabolite dynamics and regulation into the many strain design tools created around FBA. Knowing how metabolite concentrations will change over the course of an experiment could be crucial for making more informed decisions when designing strains in metabolic engineering. Current limitations in metabolomics data acquisition such as absolute quantification of metabolite concentrations and sufficient time resolution of samples make applying this work to existing metabolomics data still challenging, but as advancements in mass spectrometry and data processing methods occur to tackle these limitations [25], modeling tools such as LK-DFBA will be ready to take full advantage of the resulting data.

Taken together, this represents the first-ever linearly-constrained modeling framework with the ability to predict metabolite dynamics and directly integrate metabolomics data. Moving beyond steady-state assumptions to address the biological realities and changing metabolite levels of systems is a key step in enabling metabolic modeling to have an even greater impact on systems biology. This unique, scalable approach to the problem of dynamic modeling, circumventing some important issues of current modeling modalities, has the potential to enable a new class of metabolic models with broad applications. Genome-scale dynamic modeling of metabolic systems is a critical grand challenge in systems biology, with its solution potentially enabling broad scientific discovery and countless engineering applications, such as strain design, gene essentiality analysis, drug targeting, and understanding disease [43, 44]. LK-DFBA has been designed with an eye towards future applications in large models, as its linear programming structure offers it the potential to efficiently solve for metabolite concentrations and fluxes at the genome-scale.

Before implementing a genome-scale model into LK-DFBA, though, there are a few points to consider. First, similar to an ODE model, the parameterization of the linear kinetics constraints is the most computationally time-consuming component of LK-DFBA. The linear kinetics constraints consist of two parameters each, which are fewer and less time-consuming to solve for than parameters in most ODE models that do not just use simple Michaelis-Menten kinetics. Nevertheless, as more reactions are added to the system, the time it takes to solve for an optimal set of parameters increases, and faster optimization approaches will need to be considered. Once the parameters are calculated, the time it takes to solve for metabolite concentrations and fluxes is on par with FBA. We note, though, that the LK-DFBA (LR) method without optimization (which requires very little computational time to estimate parameters) has been shown to be comparable to other ODE-based approaches at realistic conditions (Fig. 3c), supporting the potential for viable scale-up. Second, one novel aspect of LK-DFBA is that it uses metabolite-dependent regulation as a means to increase model accuracy. In the case of genome-scale models, many of these metabolite-dependent regulations may be unknown in the literature and must be identified by other approaches in order to maintain the effectiveness of LK-DFBA. In the absence of this, optimization of the regulatory structure based on metabolomics could be possible, though we expect that to be a challenge entailing significant future effort. Third, because LK-DFBA relies on flux data to optimize the linear kinetics constraint parameters, the development of improved analytical tools for measuring fluxomics data or methods for accurately calculating flux from concentration data in underdetermined systems will significantly improve the predictions of LK-DFBA. A systematic approach to DFE for underdetermined systems by Chou et al. has produced promising results [40].

In the work discussed here, we modeled regulatory kinetics constraints that correspond to regulation of fluxes via rapid, direct mechanisms such as allostery. However, LK-DFBA is not inherently restricted to modeling this type of regulation. By choosing a simulation interval over which transcriptional changes are relevant, changes in enzyme levels could easily be modeled as well, though changes in target fluxes associated with transcriptional regulation may need to be implemented with a time delay due to the intermediate biochemical steps necessary to produce the relevant changes in enzyme levels. However, in the case of extremely sparsely sampled time courses, even a time delay might not be necessary to indirectly represent regulation by metabolites mediated through transcription.

## Conclusion

Currently, metabolic modeling frameworks are restricted to modeling the dynamics of small-scale systems or only model genome-scale systems at steady state. However, the metabolism of an organism is heavily regulated and its metabolic state is likely to change over time. Without being able to model metabolite dynamics in the entirety of an organism, metabolic engineering efforts will inevitably come up short. Development of genome-scale models with the ability to track metabolite dynamics and account for regulation will enable more accurate prediction and more effective metabolic engineering that could have a drastic impact on titers and productivity. Our work here establishes a basis for working towards this goal, and merits further investigation to see such applications to fruition.

## Supplementary information

**Additional file 1.** Supplementary Information. Contains supplementary methods, tables, and figures.

## Data Availability

The code and datasets generated during the current study are available at https://github.com/gtstylab/lk-dfba.

## Abbreviations

BST: Biochemical Systems Theory
CBM: Constraint-based Model
CoV: Coefficient of Variance
DFBA: Dynamic Flux Balance Analysis
DFE: Dynamic Flux Estimation
DOA: Dynamic Optimization Approach
FBA: Flux Balance Analysis
LK-DFBA: Linear Kinetics-Dynamic Flux Balance Analysis
LP: Linear Program
LR: Linear Regression
MM: Michaelis-Menten
ODE: Ordinary Differential Equation
PPP: Pentose Phosphate Pathway
prSSE: Penalized Relative Sum of Squares Error
QP: Quadratic Program
SOA: Static Optimization Approach
