# Supplementary material for: LK-DFBA: a linear programming-based modeling strategy for capturing dynamics and metabolite-dependent regulation in metabolism
Source: BMC Bioinformatics. 2020 Mar 2;21:93. doi: 10.1186/s12859-020-3422-0 (PMC7053146; doi:10.1186/s12859-020-3422-0)
Supplement: Supplementary file 1 — Additional file 1. Supplementary Information. Contains supplementary methods, tables, and figures. [file 12859_2020_3422_MOESM1_ESM.pdf]

Supplementary Information

LK-DFBA: A Linear Programming-based modeling strategy for capturing dynamics and metabolite-dependent regulation in metabolism

Robert A. Dromms; Justin Y. Lee; Mark P. Styczynski

Table of Contents

1. Methods S1 .... **2**
1.1 Simulating Regulated Metabolite Dynamics with a Linearly-Constrained Program 2
1.1.1 Model Input 2
1.1.2 Discretizing the Time Interval 2
1.1.3 Stoichiometry and Pooling Fluxes 2
1.1.4 Difference Equations 3
1.1.5 The Solution Vector 3
Fig. S1. A graphical depiction of the LK-DFBA modeling framework 4
1.1.6 Constant Constraints on Concentration and Flux Values 5
1.1.7 Linearized Kinetics Constraints 5
1.1.8 Model Objective 7
1.1.9 The LK-DFBA Optimization Problem 7
1.2 Test Models 7
1.2.1 The Branched Pathway Model 8
Table S1. Parameters used to generate noise-free branched pathway data sets 8
1.2.2 Glycolysis and Pentose Phosphate Pathway in E. coli 9
Fig. S2. The model of *E. coli* central carbon metabolism 10
Table S2. Metabolite abbreviations used in the *E. coli* model 11
Table S3. Flux abbreviations used in the *E. coli* model 12
1.3 Parameter Fitting 13
1.3.1 Parameter Optimization 13
Fig. S3. Bounding the parameter search space for the Genetic Algorithm 14
1.3.2 Michaelis-Menten ODE Model Rate Laws and Parameters 15
2 Results S1 .... **16**
2.1 Simulating a Time Course with a Nominal Set of Parameters 16
Fig. S4. Examples of time course simulations using LK-DFBA 17
Fig. S5. Inclusion of pooling fluxes alone is insufficient to enable meaningful metabolite dynamics 18
Fig. S6. The terminal objective was prone to several serious numerical deficiencies 19
Fig. S7. Qualitative comparison of solution-norm penalization schemes 20
2.2 Assessment of Five Model Types on Noiseless Branched Pathway Data 21
Fig. S8. Quantitative comparison of prSSE for the BST, MM, LK-DBA (LR), LK-DFBA (LR+), and LK-DFBA (GA) methods for 15 parameterizations of the branched pathway model. 21
2.3 The Effects of Withholding Metabolite Time Courses on LK-DFBA Models Performance in the Branched Pathway Model 22
Fig. S9. Comparing error contribution for Missing-X1 and Missing-X2 cases 22
2.4 Recapitulating Results with the E. coli Model 23
Fig. S10. Dynamic Flux Estimation in the *E. coli* model 23
2.5 Parameter Sensitivity of LK-DFBA in Branched Pathway Model 24
Fig. S11. Parameter sensitivity of LK-DFBA (LR+) method across different nT and CoV combinations 24
3 References **25**

1. Methods S1

1.1 Simulating Regulated Metabolite Dynamics with a Linearly-Constrained Program

1.1.1 Model Input

We describe here the implementation of our modified form of dynamic flux balance analysis (DFBA) [1]. It takes as input two sets of information. The first set comprises the constraints and objective from FBA: a stoichiometric matrix describing the relationship between metabolites and fluxes in the model, a set of upper and lower bounds on metabolic fluxes, and an objective function specifying the flux(es) the network tries to locally maximize or minimize. To these, we add metabolite concentration initial conditions, a time interval, a parameter describing the number of segments into which the simulation interval is to be evenly divided, and a list of regulatory interactions (and the corresponding parameters to describe them).

We developed a procedure in MATLAB to automatically translate a standard FBA model into an LK-DFBA model, and then solve the resulting optimization problem. This procedure works by taking as input the original FBA model, plus the inputs specified above. This code has been made publicly available on GitHub at https://github.com/gtStyLab/lk-dfba.

1.1.2 Discretizing the Time Interval

Following the basic template of DFBA [1], the simulation interval is divided into nT segments, as shown in Fig. S1E. By our convention, metabolite concentrations are represented at the time points separating the intervals, and fluxes are represented over the interval between time points. Initial conditions for metabolite concentrations specify the concentrations at the time point prior to the first interval.

1.1.3 Stoichiometry and Pooling Fluxes

The mass balances on a set of n_m metabolites, $\vec{x}$, can be represented by the system of equations

$$\frac{d\vec{x}}{dt} = S\vec{v}$$

where $\frac{d\vec{x}}{dt}$ is the accumulation or depletion of metabolites in the system, S is the stoichiometric matrix describing the connectivity of n_m metabolites and n_v fluxes in the metabolic network, and $\vec{v}$ is a vector of the n_v enzymatic rates through the metabolic network, i.e. the flux distribution.

In FBA, $\frac{d\vec{x}}{dt}$ is assumed to be zero, and the linear equation

$$0 = S\vec{v}$$

applies. Combined with a linear objective function

$$z = \vec{c}^{T}\vec{v}$$

where c_i specifies the weight of flux v_i in the objective (e.g. to maximize growth rate, set so that $c_{biomass} = 1$ and all other $c_i = 0$) and bounds

$$\vec{v}_{LB} \le \vec{v} \le \vec{v}_{UB},$$

an LP can be specified as

$$\max_{\vec{v}} z = \vec{c}^T\vec{v}$$
$$\text{s.t.}\, 0 = S\vec{v}$$
$$\vec{v}_{LB} \le \vec{v} \le \vec{v}_{UB}.$$

In LK-DFBA, we relax the steady-state assumption, working from

$$\frac{d\vec{x}}{dt} = S\vec{v}$$

Moving the $\frac{d\vec{x}}{dt}$ term to the right side and adding it to the solution vector, gives us the system shown in Fig. S1B,

$$0 = A\vec{w} = [S \quad -I]\begin{bmatrix}\vec{v}\\ \vec{v}_p\end{bmatrix}$$

where A is the (n_m × (n_m+n_v)) augmented stoichiometric matrix and $\vec{w}$ is the ((n_m+n_v) × 1) augmented flux vector, and using the "pooling flux" nomenclature of Covert *et al.* in iFBA [2],

$$\vec{v}_p = \frac{d\vec{x}}{dt}$$

i.e., we will describe $\frac{dx_i}{dt}$ as the pooling flux $\vec{v}_{p,i}$ for metabolite x_i. This augmented stoichiometric constraint will apply over each segment of the discretized interval, producing a set of $n_m \cdot nT$ constraint equations,

$$0 = A\vec{w}(t_k)$$

where $\vec{w}(t_k)$ is the augmented flux vector $\vec{w}$ evaluated at the interval ending at t_k and nT is the number of segments into which the overall time interval has been discretized.

1.1.4 Difference Equations

Concentrations are explicitly represented in the model, and metabolite dynamics are incorporated by integrating metabolite concentrations over each interval using a difference equation and the corresponding pooling flux term (i.e. the $\frac{dx_i}{dt}$ term)

$$x_i(t_k) = x_i(t_k - 1) + \Delta t \cdot v_{p,i}(t_k)$$

to produce a series of $n_m \cdot nT$ constraint equations, as shown in Fig. S1E.

1.1.5 The Solution Vector

Combining the augmented flux vector over each time segment and the concentrations at each time point, the final solution vector for the LP is constructed as

$$\omega = [\vec{w}^T(t_1), \vec{w}^T(t_2), \ldots \vec{w}^T(t_{nT-1}), \vec{w}^T(t_{nT}), \vec{x}^T(t_0), \vec{x}^T(t_1), \ldots \vec{x}^T(t_{nT-1}), \vec{x}^T(t_{nT})]^T$$

and is of dimension ((n_v + n_m) · nT + n_m · (nT + 1) × 1).

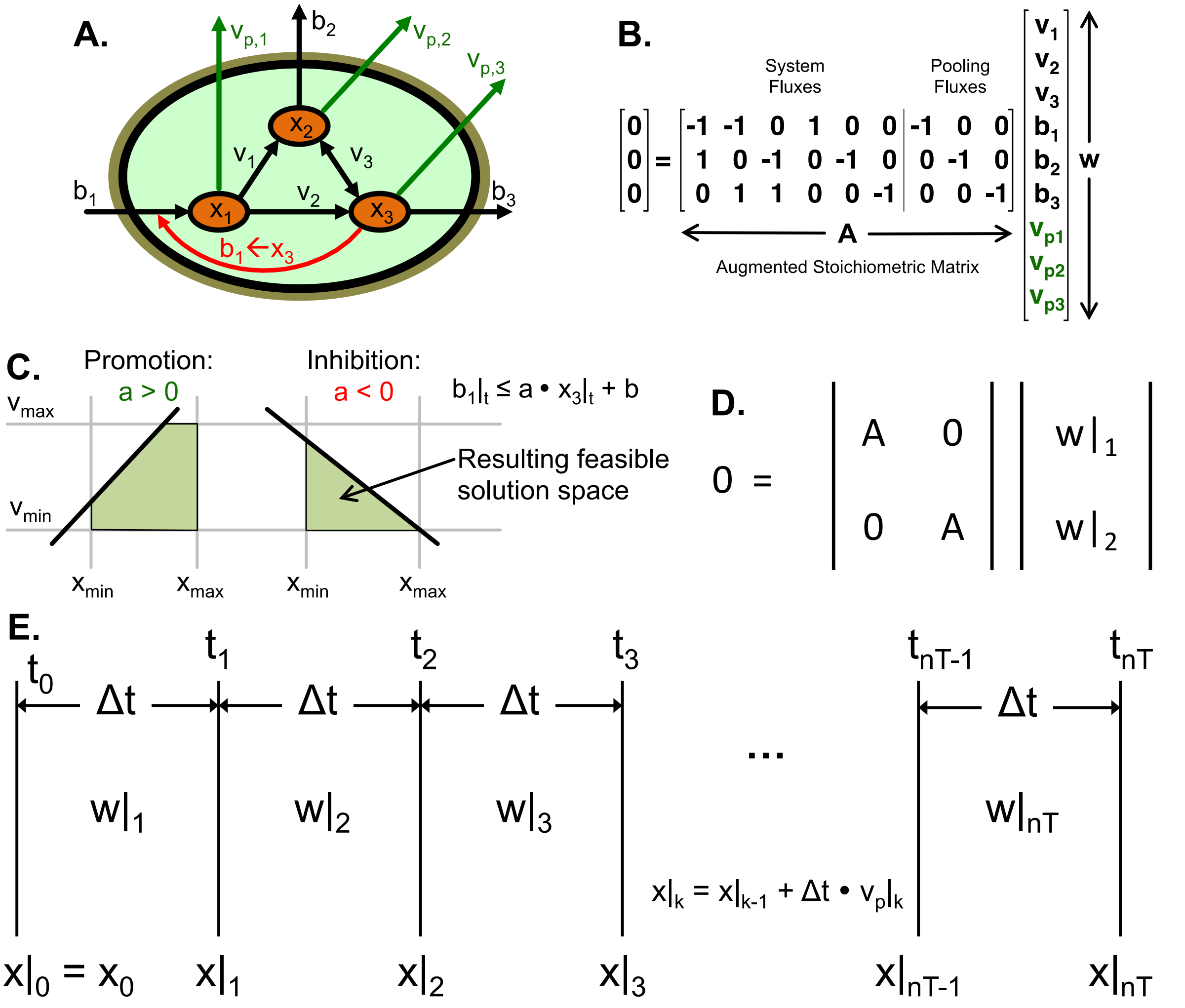

Fig. S1. A graphical depiction of the LK-DFBA modeling framework
(A) Pooling fluxes are added to capture model [3] accumulation
(B) Model stoichiometry is adjusted to include pooling fluxes
(C) Linear constraints describe positive and negative regulation dependent on metabolite concentrations
(D) Constraints can be systematically generated as templates applied across time steps
(E) The time course is discretized and accumulation integrated over time steps

1.1.6 Constant Constraints on Concentration and Flux Values

As in FBA, lower and upper bounds on system fluxes are provided and apply to the flux distribution at each interval. Typically, the upper bound constraints on irreversible internal system fluxes (and both bounds on reversible reaction fluxes) are expected to be inactive and are set at a large nominal value to guarantee the space is bounded. If upper bounds on system fluxes are known, they can be implemented into the constraints and represent saturation. Pooling fluxes are given nominal bounds as well; due to limitations on the product $\Delta t \cdot v_p$ combined with constraints on concentrations and elsewhere in the system, it is expected that the nominal bounds will not act as active constraints.

Like bounds on flux values, constraints bounding concentrations can be set by the user, but generally it is expected that concentrations are strictly positive. If a concentration is known to be at a fixed quantity, the upper and lower bound can be set accordingly.

The initial conditions $\vec{x}_0$ are specified by setting $\vec{x}(t_0) = \vec{x}_0$.

1.1.7 Linearized Kinetics Constraints

The key feature of LK-DFBA is the addition of linear equations to describe constraints in which fluxes are controlled by metabolites, as is the case in circumstances ranging from mass action kinetics to allosteric regulation (on short time scales) or transcriptional regulation (on longer time scales). Any dependence of flux on metabolite concentrations is implemented in this manner, and this turns out to be a critical requirement for enabling relevant dynamics in the model.

These constraints are specified by a list of n_r mappings. Corresponding to each mapping is a pair of parameters (a, b) such that for mapping n between "controller" metabolites $\{x\}_n$ and "target" fluxes $\{v\}_n$,

$$\sum_i v_{i,n}(t_{k+1}) \leq a_n\left(\sum_j x_{j,n}(t_k)\right) + b_n,$$

where $v_{i,n}$ is a target flux in $\{v\}_n$, $x_{j,n}$ is a controller metabolite in $\{x\}_n$, and (a_n, b_n) are the parameters describing the linear kinetics constraint. When $a_n > 0$, this interaction produces a promotional effect, and when $a_n < 0$, this interaction has an inhibitory effect. Applied over the whole discretized time course, this produces a total of $n_r \cdot nT$ kinetics constraint equations. Examples of these mappings are shown in Fig. S1C.

These constraints allow us to not only represent interactions such as allosteric regulation, but also to linearly approximate the dependence of enzyme activity on its substrate concentration. Consider the case of the positive regulator shown in Fig. S1C: we note that the profile produced by simultaneously considering the effect of the constant flux bounds constraints ("v_{max}") in conjunction with the constraint produced by mapping the enzyme substrate as a "regulator" of enzyme flux $v \leq a \cdot x + b$ is comparable to the flux vs concentration profile of a simple Michaelis-Menten reaction mechanism. We refer to these types of "kinetics" constraints as "mass action" constraints, to differentiate them from "regulatory" constraints produced through mechanisms such as allostery. Our code includes a procedure to automatically generate mass action kinetics constraints from a

stoichiometric matrix, giving the user the convenience of only needing to manually specify the regulatory constraints.

We note that these regulatory interactions are implemented as bounds on the controlled fluxes, rather than as equality constraints: this is a very fundamental difference from the behavior of ODE models, in which each equation reduces the dimension of the solution space. For linear equations, this would often create cases in which the system of equations would produce negative concentrations, for example by forcing an efflux term to exceed an influx term when a metabolite was already depleted. However, in LK-DFBA, this just leads to a situation in which the kinetics constraint is no longer active, and the other model constraints preclude blatantly unphysical behavior. As a result, LK-DFBA has a degree of both simplicity and flexibility that comes with advantages and disadvantages that we will explore in more depth in the Results S1 section.

In a model with n_r regulatory constraints, each regulatory constraint adds two parameters (a, b) to the model, for a total of $(2 \cdot n_r)$ parameters. In many cases, a single controller is paired with a single target, but certain cases may allow lumping multiple species together, allowing a reduction in the number of model parameters. For example, one might reduce the number of model parameters by choosing to constrain only the sum of the effluxes from a given metabolite, such as at branch point, rather than to introduce separate constraints for each of the individual fluxes. Our Results and Discussion includes an assessment of the tradeoffs between these two options to determine if this consolidation represents an improvement or an oversimplification.

In the work discussed here, we modeled regulatory kinetics constraints that correspond to regulation of fluxes via rapid, direct mechanisms such as allostery. However, LK-DFBA is not inherently restricted to modeling this type of regulation. By choosing a simulation interval over which transcriptional changes are relevant, changes in enzyme levels could easily be modeled as well. Capturing these other types of regulation may require modifications to reflect differences in the underlying mechanisms. For example, changes in target fluxes associated with transcriptional regulation may be subject to a time delay due to the intermediate biochemical steps necessary to produce the relevant changes in enzyme levels. Such a time delay could be introduced by shifting the linkage between controller metabolites and target fluxes from the adjacent time interval to instead a later time interval, with the exact offset specified using parameters set by the user or determined through parameter fitting.

To run a given simulation, the set of (a, b) parameter values is provided along with the map of controllers and targets. In practice, this will need to be determined via parameter fitting, as the linear equations in general are simplified approximations that do not directly correspond to intrinsic physical quantities. We consider this question in-depth in subsequent sections, where we provide several methods for determining these parameters and comment on their effectiveness and practicality.

1.1.8 Model Objective

As in FBA, LK-DFBA requires an objective. While there are several ways in which to construct this objective[37] we found that an objective that applied to the fluxes weighed equally at each time point (an "instantaneous" objective) was effective in producing stable, robust behavior. This style of objective function can be generated easily by expanding the original FBA objective to apply over each interval. We also tested an alternate "terminal" objective function (in which the objective function only looks at the concentration of a selected final time point), and found that this often led to degenerate solutions, erratic behavior, and numerical artifacts at intermediate time points. We discuss this more in the Results S1 section.

We also found it effective to add a modest penalty to the L_2 norm of the solution vector during optimization, changing the problem objective to

$$z = \vec{c}^T\vec{\omega} + \vec{\omega}^T Q\vec{\omega}$$

where

$$Q = -\lambda I$$

and λ is a small penalty on the solution norm. This has the effect of imposing a parsimony preference on the solution vector $\vec{\omega}$, which has been shown to be an effective and reasonable strategy, and helped resolve some occasional observed issues with solution degeneracy [4]. While the resulting problem is technically now a (linearly-constrained) quadratic program (QP), we observed no appreciable increase in solution time, and this particular case still specifies a convex optimization (and therefore preserves the very desirable Strong Duality features of the LP). The option to instead use the linear objective remains, but our implementation defaults to the QP formulation.

1.1.9 The LK-DFBA Optimization Problem

Assembling the constraints described in the previous sections produces the following linearly-constrained QP for simulating metabolic time courses which we refer to as LK-DFBA. For

$$\vec{\omega} = [\vec{w}^T(t_1), \vec{w}^T(t_2), \ldots \vec{w}^T(t_{nT-1}), \vec{w}^T(t_{nT}), \vec{x}^T(t_0), \vec{x}^T(t_1), \ldots \vec{x}^T(t_{nT-1}), \vec{x}^T(t_{nT})]^T,$$

$$\max_{\vec{\omega}} z = \vec{c}^T\vec{\omega} - \lambda\vec{\omega}^T\vec{\omega}$$

$$\begin{aligned}
\text{s.t.}\quad & 0 = A\vec{w}(t_k) && \forall\, k \in [1, nT] \\
& \vec{w}_{LB} \le \vec{w}(t_k) \le \vec{w}_{UB} && \forall\, k \in [1, nT] \\
& \vec{x}_{LB} \le \vec{x}(t_k) \le \vec{x}_{UB} && \forall\, k \in [1, nT] \\
& \vec{x}(\mathrm{t}_0) = \vec{x}_0 \\
& x_i(t_k) = x_i(t_{k-1}) + \Delta t \cdot v_{p,i}(t_k) && \forall\, k \in [1, nT] \\
& \sum_i v_{i,n}(t_{k+1}) \le b_n + a_n \sum_j x_{j,n}(t_k) \\
& \qquad \forall\, k \in (1, nT), \forall\, i \in \{v\}_n, \forall\, j \in \{x\}_n, \forall\, n \in (1, n_r)
\end{aligned}$$

1.2 Test Models

To test our modeling and parameter fitting strategies, we used several models to produce "synthetic" datasets. The advantage of using these datasets as a point of comparison is

that it allows us to produce idealized cases under which we can study the theoretical performance and limitations of our modeling strategy without being limited by practical concerns such as data sampling frequency, signal-to-noise ratio in the data, or limits in the cross-section of metabolites we can measure.

1.2.1 The Branched Pathway Model

Our first test model is a modified version of a popular, well-established *in silico* model from Biochemical Systems Theory (BST) describing a simple branched pathway with both positive and negative regulatory interactions [5]. Our modified version introduces several changes and is shown in Fig. 1 of the main text.

Table S1. Parameters used to generate noise-free branched pathway data sets

	Stoichiometry		Kinetics											Initial Conditions				
k	bm_3	bm_4	a_2	b_{21}	a_3	b_{32}	b_{r34}	a_4	b_{41}	b_{r43}	a_5	b_{53}	b_{54}	X_1	X_2	X_3	X_4	X_{BM}
1	0.6	0.4	0.8	0.5	1.0	0.8	0.2	0.5	0.4	-0.8	0.5	0.5	0.8	0.1	0.2	0.3	0.4	0.5
2	0.6	0.4	0.8	0.5	1.0	0.8	0.2	0.5	0.4	-0.8	0.5	0.5	0.8	0.1	0.1	0.1	0.1	0.1
3	0.6	0.4	0.8	0.5	1.0	0.8	0.2	0.5	0.4	-0.8	0.5	0.5	0.8	1.0	1.0	1.0	1.0	1.0
4	0.6	0.4	0.22	0.925	0.691	0.856	0.302	0.416	0.107	-0.564	0.436	0.816	0.52	1.0	1.0	1.0	1.0	1.0
5	0.6	0.4	0.935	0.457	0.24	0.763	0.759	0.74	0.743	-0.106	0.681	0.463	0.212	1.0	1.0	1.0	1.0	1.0
6	0.6	0.4	0.52	0.725	0.791	0.656	0.402	0.816	0.807	-0.364	0.936	0.616	0.82	1.0	1.0	1.0	1.0	1.0
7	0.6	0.4	0.679	0.036	0.809	0.748	0.12	0.525	0.325	-0.546	0.398	0.415	0.18	1.0	1.0	1.0	1.0	1.0
8	0.9	0.1	0.8	0.5	1.0	0.8	0.2	0.5	0.4	-0.8	0.5	0.5	0.8	1.0	1.0	1.0	1.0	1.0
9	0.8	0.2	0.8	0.5	1.0	0.8	0.2	0.5	0.4	-0.8	0.5	0.5	0.8	1.0	1.0	1.0	1.0	1.0
10	0.7	0.3	0.8	0.5	1.0	0.8	0.2	0.5	0.4	-0.8	0.5	0.5	0.8	1.0	1.0	1.0	1.0	1.0
11	0.5	0.5	0.8	0.5	1.0	0.8	0.2	0.5	0.4	-0.8	0.5	0.5	0.8	1.0	1.0	1.0	1.0	1.0
12	0.4	0.6	0.8	0.5	1.0	0.8	0.2	0.5	0.4	-0.8	0.5	0.5	0.8	1.0	1.0	1.0	1.0	1.0
13	0.3	0.7	0.8	0.5	1.0	0.8	0.2	0.5	0.4	-0.8	0.5	0.5	0.8	1.0	1.0	1.0	1.0	1.0
14	0.2	0.8	0.8	0.5	1.0	0.8	0.2	0.5	0.4	-0.8	0.5	0.5	0.8	1.0	1.0	1.0	1.0	1.0
15	0.1	0.9	0.8	0.5	1.0	0.8	0.2	0.5	0.4	-0.8	0.5	0.5	0.8	1.0	1.0	1.0	1.0	1.0

First, we replaced the two effluxes in the original model with a single, fixed-stoichiometry “biomass” reaction, which produces a biomass “metabolite” subject to a mass balance equation. This introduces some additional biological relevance (such reactions are ubiquitous in genome-scale models) and allows us to define a clear objective for the system.

Second, we modified the two regulatory interactions to change their targets. This allows us to simplify the model while still producing interesting dynamics for X_1 via interactions with the branch fluxes v_2 and v_4. Originally, the negative feedback regulator controlled by metabolite X_3 targeted the system input flux, v_1. We changed its target from v_1 to the branch flux of the opposing lower pathway, v_4. We then left the input flux v_1 at a constant value of v_{in}. We also changed the positive regulatory interaction controlled by X_4 to instead target v_3, the first flux in the opposing upper branch. This allowed us to avoid introducing a parameter identifiability problem when X_4 acts as a controller for flux v_5 via both mass action kinetics and regulation (as would have been the result of combining the two branch outlet fluxes into a single biomass composition reaction).

Like the original BST model, we implement power-law kinetics, as shown in the equations of Fig. 1 of the main text. We produce several noiseless datasets by modifying the initial

conditions, biomass equation stoichiometry, and kinetic rate constants; the conditions for these models are shown in Table S1.

1.2.2 Glycolysis and Pentose Phosphate Pathway in E. coli

While the branched pathway model is convenient as an initial test case, it lacks physiological significance and is too simple to capture some of the challenges we expect in real metabolic networks. To explore initial scale up and to better gauge the challenges of implementing LK-DFBA, we test a model of central carbon metabolism in *Escherichia coli*, specifically encapsulating glycolysis and the pentose phosphate pathway (PPP) [6]. The network structure is shown in Fig. S2, and a list of model abbreviations in Table S2 and S3. The model is a system of ODEs with empirically derived rate laws. Metabolite concentrations were generated using the procedure described by Dromms and Styczynski [7]. Briefly, noiseless data at high resolution were generated from the default model initial conditions and parameters in Copasi 4.14 (Build 89), with the exception that moieties such as ATP, ADP, and NADH, etc. were held at constant concentrations during simulation [6-10].

Reversible reactions were implemented by splitting them into two irreversible reactions representing the forward and reverse directions. For mass action constraints, the substrate of the original reaction was designated as the controller for the forward reaction, and the product was set as the controller for the reverse reaction. Fluxes such as Met and Trp synthesis, which are set to fixed values in the ODE model, were explicitly provided this information as well. We modeled the degradation reactions using equality constraints where the a parameter was known and assigned to the model based on the value in the underlying ODE model, and set $b = 0$ to produce a first-order kinetic rate law. By setting $b = 0$, we avoid creating the potential for our linear kinetics constraints to create conflicts with non-negative concentration constraints that would result in an infeasible LP.

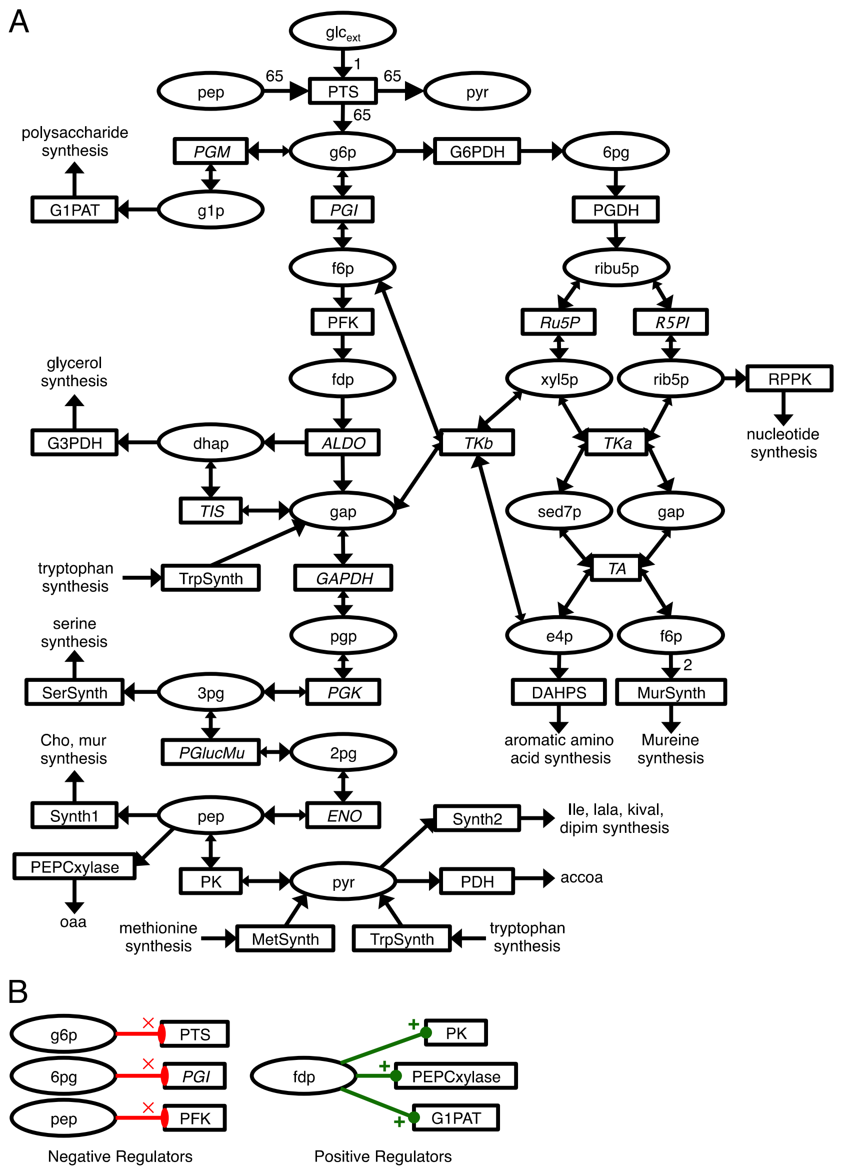

Fig. S2. The model of *E. coli* central carbon metabolism
Adapted from Figure 2 of Chassagnole *et al*. [6]. Abbreviations used in this figure are expanded in Table S2 and S3.
(A) The model includes glycolysis and the pentose phosphate pathway. Circles denote metabolites, and rectangles denote fluxes. Arrows with one head are irreversible reactions, and those with two heads are reversible; the larger head indicates the forward reaction direction. Numbers next to arrows denote non-unity stoichiometric coefficients. Not shown are degradation and dilution reactions.
(B) The regulatory connections used in our implementation of the *E. coli* model. Red 'x' connections signify negative regulators, and green '+' connections represent positive regulators.

Table S2. Metabolite abbreviations used in the *E. coli* model

Index	Metabolite	Abbreviation
1	Extracellular Glucose	glc
2	Glucose-6-Phosphate	g6p
3	Fructose-6-Phosphate	f6p
4	Fructose-1,6-bisphosphate	fdp
5	Glyceraldehyde-3-Phosphate	gap
6	Dihydroxyacetonephosphate	dhap
7	1,3-diphosphophosphoglycerate	pgp
8	3-Phosphoglycerate	3pg
9	2-Phosphoglycerate	2pg
10	Phosphoenol pyruvate	pep
11	Pyruvate	pyr
12	6-Phosphogluconate	6pg
13	Ribulose-5-phosphate	ribu5p
14	Xylulose-5-phosphate	xyl5p
15	sedoheptulose-7-phosphate	sed7p
16	Ribose-5-phosphate	rib5p
17	Erythrose-4-phosphate	e4p
18	Glucose-1-Phosphate	g1p

Table S3. Flux abbreviations used in the *E. coli* model

Index	Name	Abbreviation	Index	Name	Abbreviation
1	Extracellular glucose kinetics	glc_kin	25	Pyruvate dehydrogenase	PDH
2	Phosphotransferase system	PTS	26	Methionine synthesis	MetSynth
3	Glucose-6-phosphate isomerase	PGI	27	6-Phosphogluconate dehydrogenase	PGDH
4	Phosphoglucomutase	PGM	28	Ribose-phosphate isomerase	R5PI
5	Glucose-6-phosphate dehydrogenase	G6PDH	29	Ribulose-phosphate epimerase	Ru5p
6	Phosphofructokinase	PFK	30	Ribose phosphate pyrophosphokinase	RPPK
7	Transaldolase	TA	31	Glucose-1-phosphate adenyltransferase	G1PAT
8	Transketolase a	TKa	32	G6P degradation	g6p_deg
9	Transketolase b	TKb	33	F6P degradation	f6p_deg
10	Mureine synthesis	MurSynth	34	FDP degradation	fdp_deg
11	Aldolase	ALDO	35	GAP degradation	dhap_deg
12	Glyceraldehyde-3-phosphate dehydrogenase	GAPDH	36	DHAP degradation	dhap_deg
13	Triosephosphate isomerase	TIS	37	PGP degradation	pgp_deg
14	Tryptophan synthesis	TrpSynth	38	PG3 degradation	pg3_deg
15	Glycerol-3-phosphate dehydrogenase	G3PDH	39	PG2 degradation	pg2_deg
16	Phosphoglycerate kinase	PGK	40	PEP degradation	pep_deg
17	Serine synthesis	SerSynth	41	Pyruvate dilution	pyr_dil
18	Phosphoglycerate mutase	PGluMu	42	PG dilution	pg_dil
19	Enolase	ENO	43	Ribu5P dilution	ribu5p_dil
20	Pyruvate kinase	PK	44	XYL5P dilution	xyl5p_dil
21	PEP carboxylase	PEPCxylase	45	SED7P dilution	sed7p_dil
22	Synthesis 1	Synth1	46	Rib5P dilution	rib5p_dil
23	Synthesis 2	Synth2	47	E4P dilution	e4p_dil
24	DAHP synthesis	DAHPS	48	GLP dilution	g1p_dil

1.3 Parameter Fitting

1.3.1 Parameter Optimization

The most general strategy is a standard parameter optimization approach. We constructed a fitness function from the weighted sum-of-squares error (SSE) between the provided data and model predictions, subject to an L_2 regularization penalty on the fitted parameters. The SSE weights are specified by the user and can be used to reflect features such as differences in scale between metabolites or heuristics to enable attempts to more effectively recapitulate the behavior of certain metabolites. These weights can potentially be applied to concentrations, fluxes, or pooling fluxes, but we only used weights on concentrations in our work. Our implementation also allows the user to specify a regularization weight and reference vector for the regularization penalty.

This SSE fitness function was used to fit FBA models for methods based on non-linear optimization. For the “Regression-Plus” method (‘LK-DFBA (LR+)’), we used the results of the Linear Regression (‘LK-DFBA (LR)’) method (described below) as an initial starting point for the Nelder-Mead simplex solver using `fminsearch()` in MATLAB. The other method (‘LK-DFBA (GA)’) used the `ga()` function in MATLAB’s Global Optimization toolbox to search using a genetic algorithm. Specifications used for `fminsearch()` and `ga()` can be found in the publicly available code on GitHub.

In the case of the genetic algorithm, we improved convergence of the algorithm by introducing constraints on the parameter search space to remove areas where we anticipated poor parameter sensitivity. These restrictions are described in Fig. S3.

For larger systems, we found that it may be more tractable to perform multiple sequential optimization problems by fixing a subset of the parameter values and switching off between optimizing different parameters at each step. We provide an option for the user to specify multiple rounds of optimization, in which individual pairs of parameters can be set as fixed or fitted for a given round of optimization. This is accomplished by specifying a design matrix in which rows represent kinetics constraints, and columns specify individual optimization rounds. If a particular kinetics constraint (parameter pair) is fixed at its initial values for a particular round, its value for the corresponding column is set to 1; otherwise, if it is to be optimized, it is set to 0. For example, we simultaneously fit all 6 kinetics constraints in a single step by setting this matrix as a (6×1) matrix of zeros. For the *E. coli* model, we chose to optimize over individual constraints (i.e. individual (a, b) parameter pairs) in sequential order until we had cycled through fitting all kinetics constraints twice.

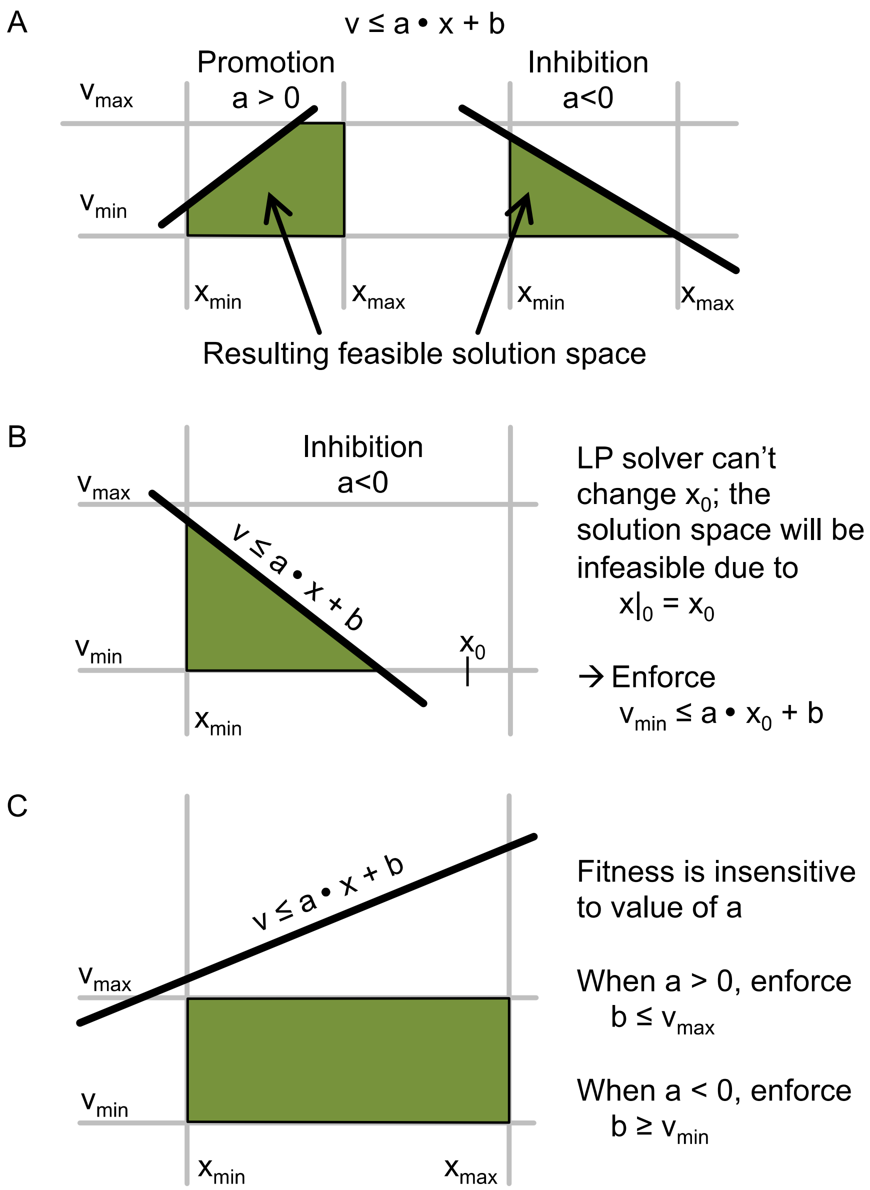

Fig. S3. Bounding the parameter search space for the Genetic Algorithm
(A) Kinetics constraints are formulated as linear inequalities. Also present are pre-specified bounds on flux values from FBA, a constraint on minimum metabolite concentrations to guarantee physically realistic concentration values, and an effective upper bound on concentration at a given time point due to mass balance constraints and the equations integrating pooling fluxes over the previous time points. This specifies a feasible solution space.
(B) Under certain regions of parameter space when $a < 0$ (inhibition), the initial concentration of the metabolite, x_0, is outside the feasible space. This produces a conflicting constraint with the kinetics constraints, and the resulting LP is infeasible. We avoid this by restricting (a, b) such that $v_{min} \leq a \bullet x_0 + b$.
(C) For certain regions of parameter space, the kinetics constraint will be guaranteed inactive. In this situation, the fitness function will be insensitive to small changes in (a, b), making it difficult to optimize these parameters. We minimize this issue by restricting b such that when $a > 0$, $b \leq v_{max}$ and when $a < 0$, $b \geq v_{min}$.

1.3.2 Michaelis-Menten ODE Model Rate Laws and Parameters

Rate law equations for the MM model were as follows:

$$v_1 = v_0$$

$$v_2 = V_2^M \frac{X_1}{V_2^K + X_1}$$

$$v_3 = \frac{V_3^M \cdot X_2}{V_3^K \left(\frac{1 + \frac{X_4}{V_3^A}}{1 + \frac{\beta \cdot X_4}{\alpha \cdot V_3^A}} \right) + X_2 \left(\frac{1 + \frac{X_4}{\alpha \cdot V_3^A}}{1 + \frac{\beta \cdot X_4}{\alpha \cdot V_3^A}} \right)}$$

$$v_4 = \frac{V_4^M \cdot X_1}{(1 + \frac{X_3}{V_4^I})(V_4^K + X_1)}$$

$$v_5 = V_5^M \frac{X_3 \cdot X_4}{V_5^K + V_{5,3}^K \cdot X_3 + V_{5,4}^K \cdot X_4 + X_3 \cdot X_4}$$

where V_2^M, V_2^K, V_3^M, V_3^K, V_3^A, α, β, V_4^M, V_4^K, V_4^I, V_5^M, V_5^K, $V_{5,3}^K$, $V_{5,4}^K$ are the fitted Michaelis-Menten parameters [11, 12]. These represent respectively a constant input flux, a single-substrate Michaelis-Menten rate law, a non-essential activation Michaelis-Menten rate law, a non-competitive inhibition Michaelis-Menten rate law, and a two-substrate Michaelis-Menten rate law.

2 Results S1

2.1 Simulating a Time Course with a Nominal Set of Parameters

We implemented LK-DFBA in MATLAB using the Gurobi solver library [13]. These codes take a model specified by the user (including an FBA model structure and the additional information for concentrations, regulation, and simulation interval, as described in the Methods section), generate the extended LP problem structure for the dynamic FBA problem, and solve the optimization using Gurobi. The results of this optimization are parsed into data matrices for the concentration and flux time course profiles, and are returned to the user. An example time course simulation is shown in Fig. S4. One behavior we observe is a change in active constraints over the time course, leading to shifts in the resulting flux distribution. We note here that an instantaneous shift in fluxes takes time to produce changes in concentrations due to the integration equations.

To demonstrate the necessity of including our linear kinetics constraints, we performed a simulation with a model containing no kinetics constraints. The result of this is shown in Fig. S5. After an initial transient period in which the metabolite pools are immediately depleted, the model quickly reverted to the steady-state flux distribution one would observe from an FBA optimization with no dynamics or regulation.

To produce the stable behavior shown in Fig. S4 and S5, we tested several options to determine the optimal configuration of the optimization problem. We explored a terminal and an instantaneous objective function, and determined that an instantaneous objective produced more stable behavior. The justification for this decision is shown in Fig. S6, in which the prevalence of degenerate solutions and inconsistent time course behavior led us to abandon the terminal objective function.

To combat degenerate solutions, we further explored penalties on the norm of the solution vector $\vec{\omega}$. These included secondary optimizations in which the optimal $z = \vec{c}^T\vec{\omega}$ was set as a constraint, and the L_1- or L_2-norm of $\vec{\omega}$ was minimized, as well as schemes penalizing $(\vec{v}(t_{k+1}) - \vec{v}(t_k))$ (not shown). The results of several regularization schemes are shown in Fig. S7. From this analysis, we concluded that the best solution was a single optimization using the instantaneous objective with a penalty on the L_2-norm of $\vec{\omega}$, which we implemented as described in the Methods as objective $z = \vec{c}^T\vec{\omega} - \lambda\vec{\omega}^T\vec{\omega}$.

In hindsight, the improved performance of the instantaneous objective function over the terminal objective is perhaps unsurprising. In a biological system, the organism lacks any foreknowledge of resource abundance, and instead is limited only to sensing the current state of its internal and external environment. The instantaneous objective better reflects this reality, and is justified both on a theoretical basis and on the practical basis demonstrated in Fig. S6 and S7.

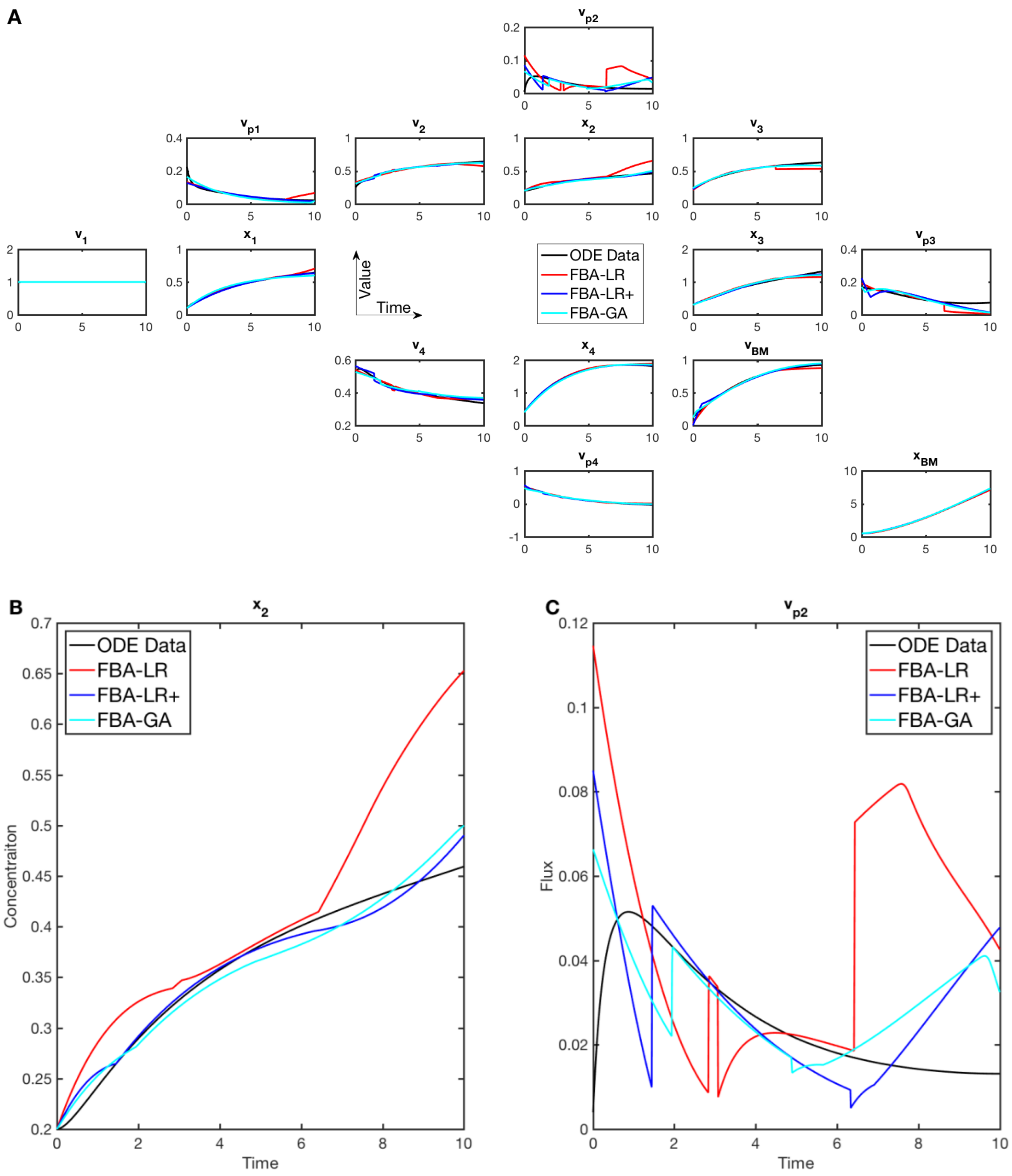

Fig. S4. Examples of time course simulations using LK-DFBA

The ODE time course data in black were fitted with the LK-DFBA (GA), LK-DFBA (LR), and LK-DFBA (LR+) methods to identify model parameters. The resulting parameters were used with the LK-DFBA model to simulate the time course behavior. All units for concentration, flux, and time are arbitrary units.

(A) The overall time course, showing metabolite concentrations, system fluxes, and pooling fluxes.

(B) Magnified view of metabolite X_2.

(C) Magnified view of pooling flux v_{p2}.

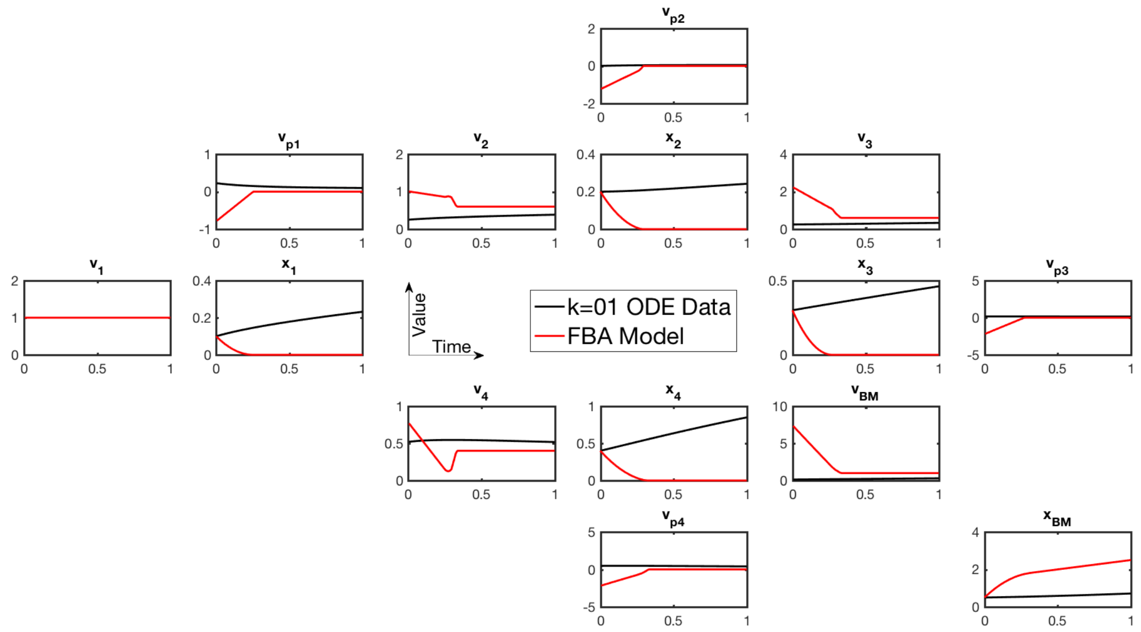

Fig. S5. Inclusion of pooling fluxes alone is insufficient to enable meaningful metabolite dynamics Additional constraints are necessary to prompt biologically relevant behavior. When the regulatory constraints are specified as an empty set, the model exhibits an initial burst of activity as the metabolite pools are consumed. This is followed by steady-state behavior in which the model produces the same steady-state flux results that are observed in an unmodified FBA model. All units for concentration, flux, and time are arbitrary units.

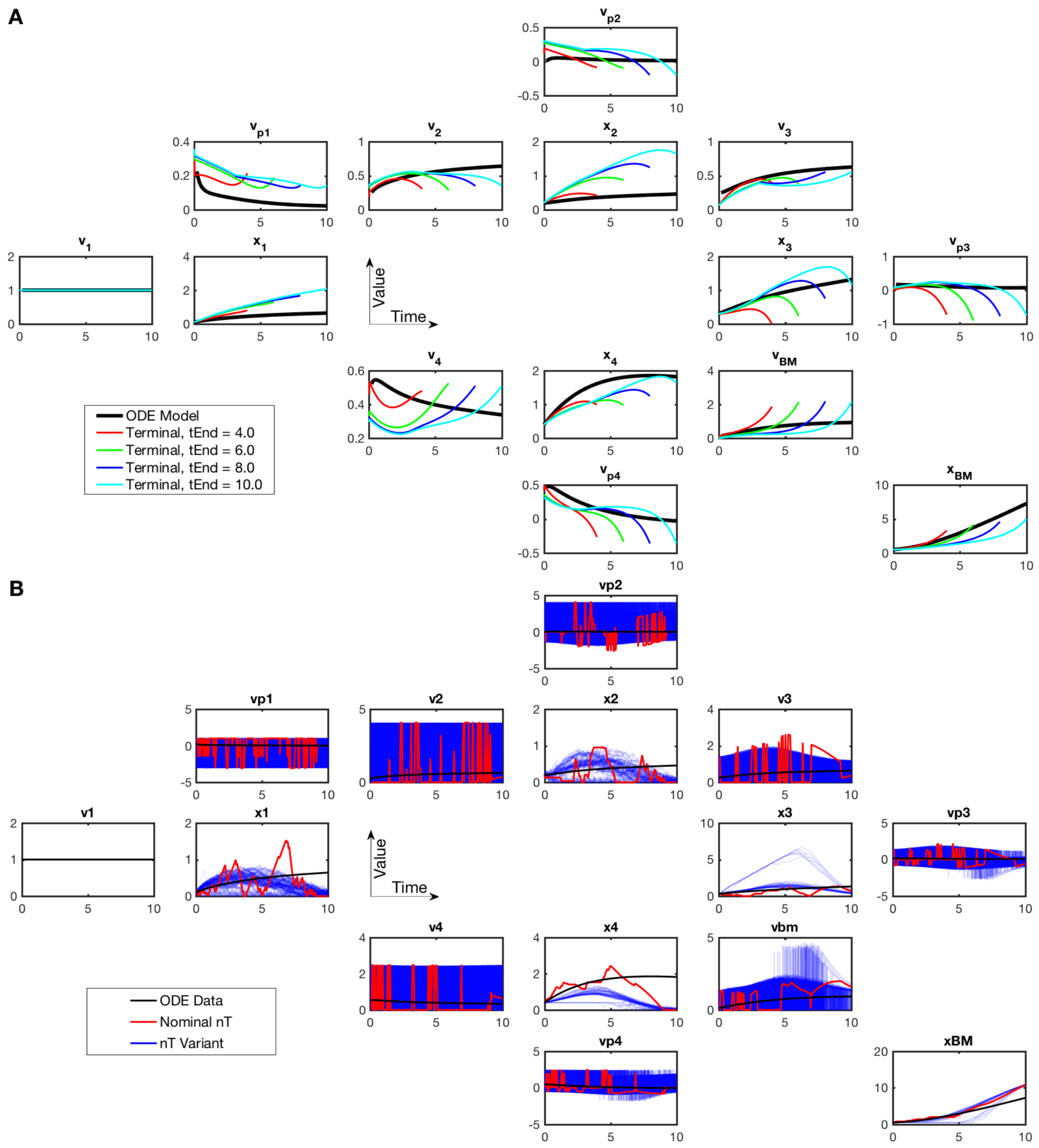

Fig. S6. The terminal objective was prone to several serious numerical deficiencies

(A) Using the same kinetics parameters and the same Δt, we simulated the model using the terminal objective under differing simulation end times. We observed that the trajectories produced for different end times were inconsistent, leading to wide variation in model behavior in the overlapping intervals. In this example, an L_2 penalty has been assigned to the solution vector to combat the degeneracy issue shown in panel B. All units for concentration, flux, and time are arbitrary units.

(B) Simulations with the terminal objective without a secondary penalty were heavily sensitive to the choice of parameter nT during simulation. In this set of graphs, the genetic algorithm was used with the terminal objective at nT = 200. The resulting parameters were simulated at nT = 200, shown in red. The simulation was repeated at nT = 150, nT = 200, nT = 250, up to to nT = 2000 with the same parameters; the resulting trajectories are shown in blue. We note that the trajectories shown here represent degenerate solutions to the optimization problem: for each trajectory, the objective function (final concentration of X_{BM}) obtains the same value.

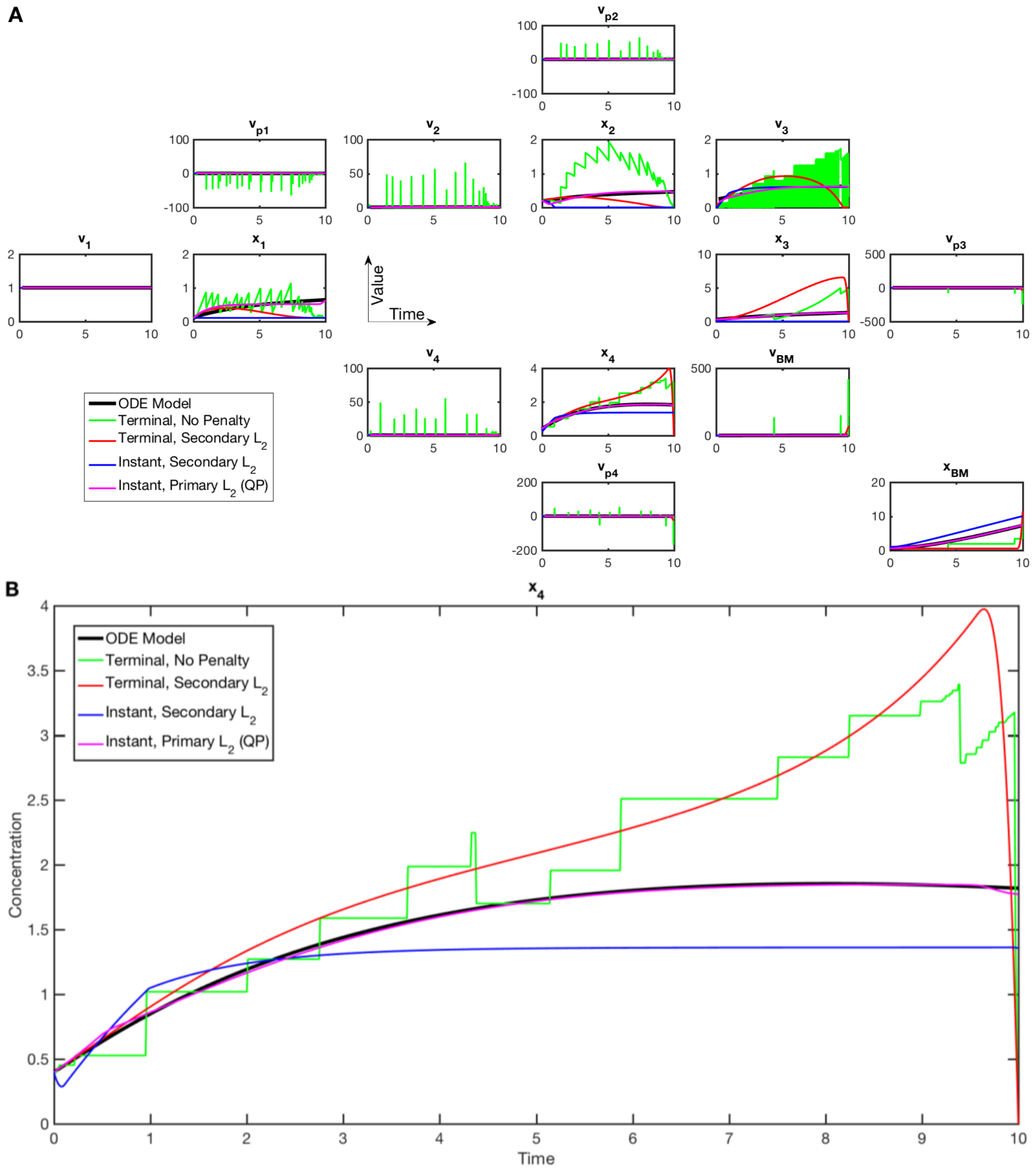

Fig. S7. Qualitative comparison of solution-norm penalization schemes

In addition to comparing the instantaneous and terminal objective types, we explored solution-norm penalization. Model trajectories were simulated from parameters identified using the genetic algorithm method and the FBA model configuration specified on high-resolution noiseless ODE data (in black). All units for concentration, flux, and time are arbitrary units.

(A) An overall comparison across the branched pathway model.

(B) A more detailed view of metabolite X_4. The un-penalized terminal objective (green) led to poor and inconsistent behavior, whereas the instant objective with primary L_2 minimization (formulated as a QP; magenta) gave the best fit and most robust behavior.

2.2 Assessment of Five Model Types on Noiseless Branched Pathway Data

As described in the Methods section, we generated a set of medium resolution (nT = 100) noiseless ODE time course profiles for the branched pathway model from the 15 parameterizations shown in Table S1. We fit models for the five methods described in the Methods section: BST, MM, LK-DFBA (LR), LK-DFBA (LR+), and LK-DFBA (GA). The fitness function for the LK-DFBA (LR+) and LK-DFBA (GA) fitting methods was configured to fit only metabolite concentrations by assigning a weight of 0 to system and pooling flux values. We simulated model dynamics at high resolution (nT = 1000) and determined the prSSE for each case; the results of this analysis are shown in Fig. S8.

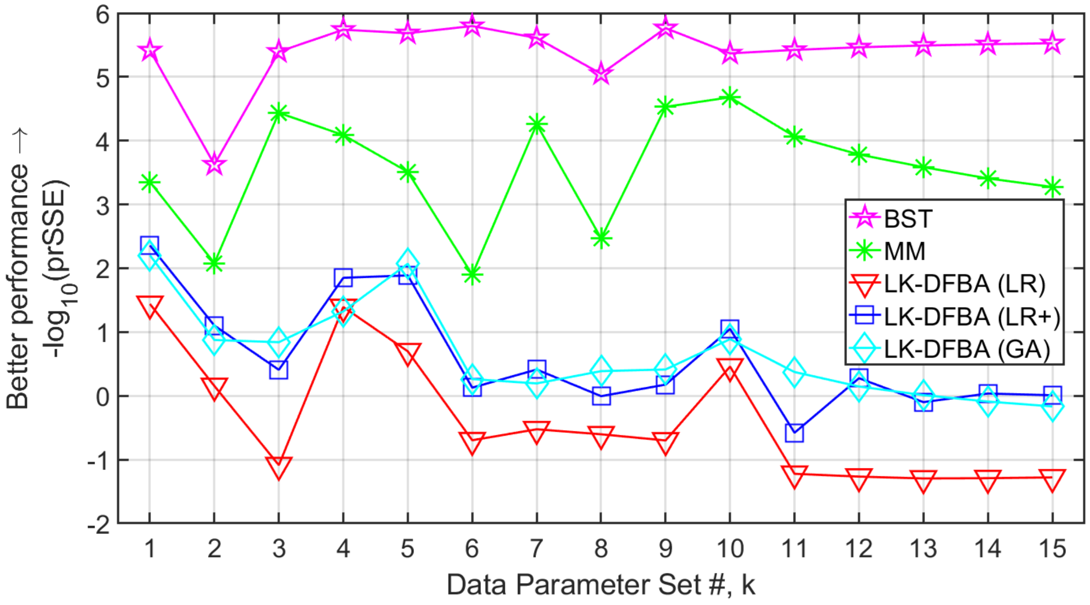

Fig. S8. Quantitative comparison of prSSE for the BST, MM, LK-DBA (LR), LK-DFBA (LR+), and LK-DFBA (GA) methods for 15 parameterizations of the branched pathway model.
Parameterizations correspond to those of Table S1. Penalized relative sum-of-square errors (prSSE) are calculated as described in the Methods section of the main text.

For the noiseless, high-resolution data sets, we observe several trends. First, the BST method has the best performance followed by the MM model. This is to be expected, since the BST method's model equations are identical to those of the underlying ODE model. Second, the LK-DFBA (LR) method has the lowest performance, which is perhaps unsurprising given the number of approximations used in this method. Third, the LK-DFBA (LR+) method substantially improves on the LK-DFBA (LR) parameters, leading it to produce time course data similar in accuracy to the LK-DFBA (GA) method. While the LK-DFBA (GA) method outperforms the LK-DFBA (LR+) method in seven of the fifteen cases, the differences are relatively small, and this modest improvement comes at the cost of 5-6 hours of computational time, compared to the <10 minutes required for the LK-DFBA (LR+) method (which in this case includes performing multiple fits with random perturbations to the initial LK-DFBA (LR) guess). For this reason, we omit using the LK-DFBA (GA) in subsequent sections.

2.3 The Effects of Withholding Metabolite Time Courses on LK-DFBA Models

Performance in the Branched Pathway Model

In the Missing-X_2 cases, the lack of data describing X_2 dynamics led to poor optimization using the LK-DFBA (LR+) method: the parameters that best optimized the remaining data pushed the model to poorly approximating the time course of the unmeasured metabolite (which was still included in the calculation of prSSE), as shown in Fig. S9. Looking at the contribution of individual metabolites to the overall error, we indeed see that the largest contribution comes from the prSSE for predicting X_2. This serves to demonstrate a point made by Goel *et al.* regarding error compensation and the advantages of performing parameter optimization over smaller independent subsets of the system via e.g. regression [14].

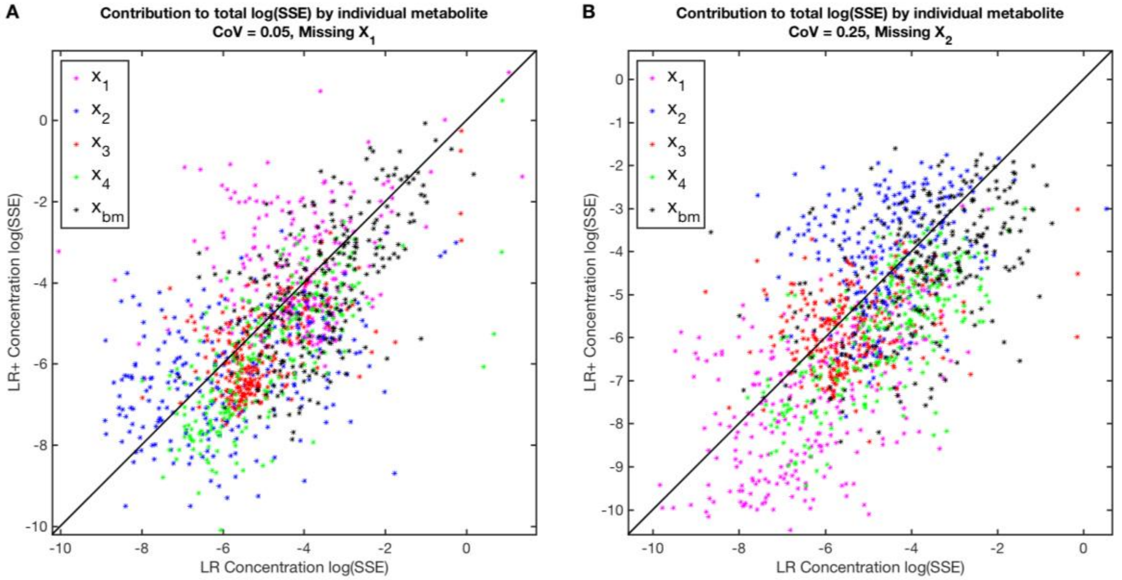

Fig. S9. Comparing error contribution for Missing-X1 and Missing-X2 cases
For a given noisy dataset, the data was fit using the LK-DFBA (LR) and (LR+) methods. The prSSE for an individual metabolite was calculated for each fitted model, and compared as shown above. The x-axis denotes the time course total prSSE of that metabolite in the LK-DFBA (LR) model, and the y-axis the corresponding prSSE in the LK-DFBA (LR+) model. Individual dots represent the error for a specific metabolite in a specific dataset, with the color indicating the metabolite. Data above the solid black line indicates that for that noisy data set, the error in the LK-DFBA (LR+) model exceeded the error in the LK-DFBA (LR) model.
(A) CoV = 0.05, Missing-X_1 data sets.
(B) CoV = 0.25, Missing-X_2 data sets.

2.4 Recapitulating Results with the E. coli Model

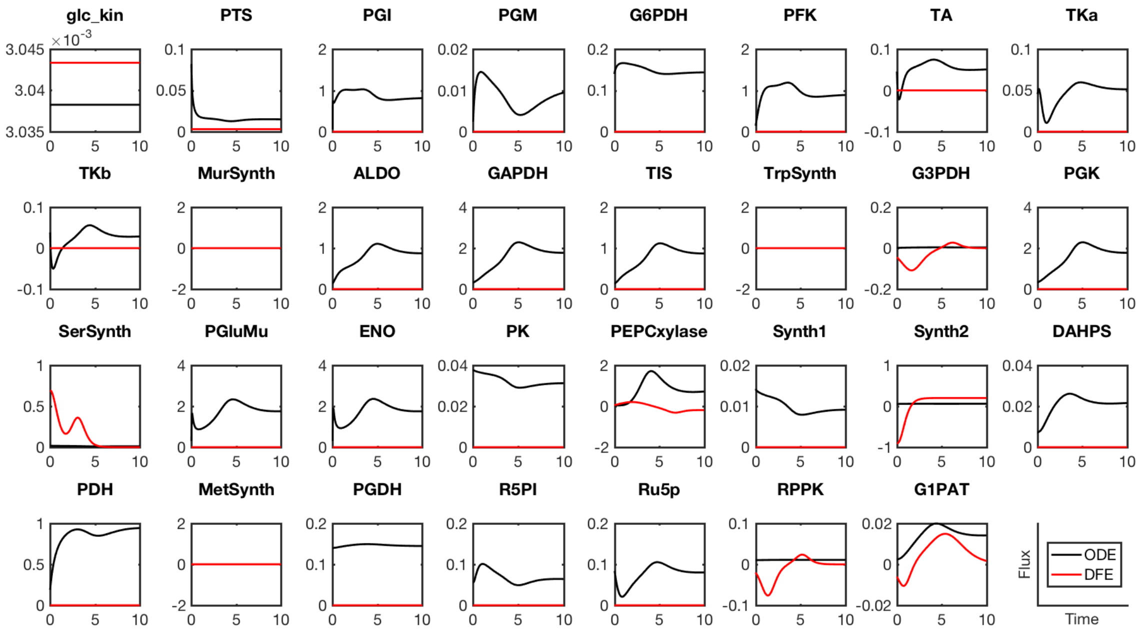

Fig. S10. Dynamic Flux Estimation in the *E. coli* model
The black trajectories are flux profiles from the ODE model, and the red trajectories are the dynamic flux distributions calculated using the DFE procedure described in the Methods section. In most cases, DFE failed to qualitatively capture the correct flux behaviors, making it difficult to use the regression method with any accuracy. We opted to instead use noise-added flux values from the ODE model for parameter regression.

2.5 Parameter Sensitivity of LK-DFBA in Branched Pathway Model

For each of the nT and CoV combinations in Fig. 3 of the main text, 50 replicate data sets were produced. We observe that some parameters in linear kinetics constraints are more sensitive than others when using the LK-DFBA (LR+) method, as shown in Fig. S11. For the branched pathway model, parameter values for a_1, a_3, a_6, b_1, b_3, and b_6 are generally stable across different nT and CoV conditions (Fig. S11A, C, F, G, I, L). Values for a_2, a_4, a_5, b_2, b_4, and b_5 are more sensitive to changes in CoV (Fig. S11B, D, E, H, J, K). For all parameters, the variance in values increases as CoV increases, which is to be expected. These results indicate that in a few key constraints it may be important to keep certain parameters within a certain range of values in order to correctly capture metabolite dynamics, whereas other parameters have more flexibility to fluctuate.

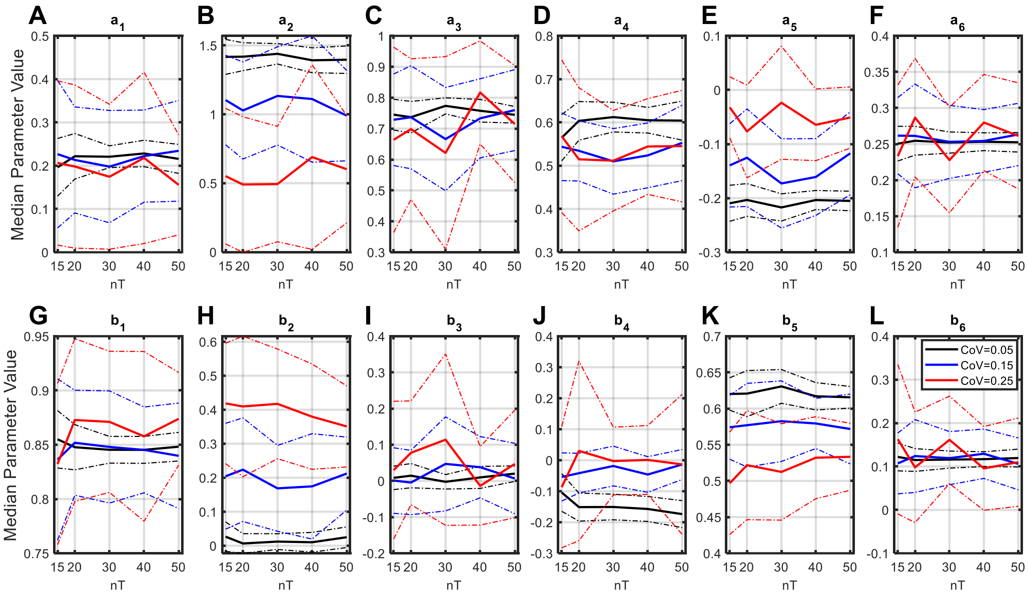

Fig. S11. Parameter sensitivity of LK-DFBA (LR+) method across different nT and CoV combinations

Thick lines represent median parameter values and thin dotted lines represent +/- the median absolute deviation.

3 References

1. Mahadevan R, Edwards JS, Doyle FJ, 3rd. Dynamic flux balance analysis of diauxic growth in Escherichia coli. Biophys J. 2002;83(3):1331-40.

2. Covert MW, Xiao N, Chen TJ, Karr JR. Integrating metabolic, transcriptional regulatory and signal transduction models in Escherichia coli. Bioinformatics. 2008;24(18):2044-50.

3. Kauffman KJ, Prakash P, Edwards JS. Advances in flux balance analysis. Curr Opin Biotechnol. 2003;14(5):491-6.

4. Lewis NE, Hixson KK, Conrad TM, Lerman JA, Charusanti P, Polpitiya AD, et al. Omic data from evolved E. coli are consistent with computed optimal growth from genome-scale models. Mol Syst Biol. 2010;6:390.

5. Voit EO, Almeida J. Decoupling dynamical systems for pathway identification from metabolic profiles. Bioinformatics. 2004;20(11):1670-81.

6. Chassagnole C, Noisommit-Rizzi N, Schmid JW, Mauch K, Reuss M. Dynamic modeling of the central carbon metabolism of Escherichia coli. Biotechnol Bioeng. 2002;79(1):53-73.

7. Dromms RA, Styczynski MP. Improved metabolite profile smoothing for flux estimation. Mol Biosyst. 2015;11(9):2394-405.

8. Hoops S, Sahle S, Gauges R, Lee C, Pahle J, Simus N, et al. COPASI--a COmplex PAthway SImulator. Bioinformatics. 2006;22(24):3067-74.

9. Ishii N, Nakayama Y, Tomita M. Distinguishing enzymes using metabolome data for the hybrid dynamic/static method. Theor Biol Med Model. 2007;4(1):19.

10. Le Novere N, Bornstein B, Broicher A, Courtot M, Donizelli M, Dharuri H, et al. BioModels Database: a free, centralized database of curated, published, quantitative kinetic models of biochemical and cellular systems. Nucleic Acids Res. 2006;34(Database issue):D689-91.

11. Strelow J DW, Iversen PW, et al. Mechanism of Action Assays for Enzymes. Bethesda (MD): Eli Lilly & Company and the National Center for Advancing Translational Sciences, 2004-; 2012 [updated 2012 Oct 1. Available from: https://www.ncbi.nlm.nih.gov/books/NBK92001/.

12. Leskovac V. Comprehensive Enzyme Kinetics. New York: Springer US; 2003.

13. Gurobi Optimization I. Gurobi Optimizer Reference Manual. 2013.

14. Goel G, Chou IC, Voit EO. System estimation from metabolic time-series data. Bioinformatics. 2008;24(21):2505-11.
